# CD226 deletion improves post-infarction healing via modulating macrophage polarization in mice

**DOI:** 10.7150/thno.37106

**Published:** 2020-01-20

**Authors:** Jun Li, Yun Song, Jing-Yi Jin, Guo-Hua Li, Yong-Zheng Guo, Hong-Yu Yi, Jin-Rui Zhang, Ya-Jie Lu, Jing-Long Zhang, Cong-Ye Li, Chao Gao, Lu Yang, Feng Fu, Fu-lin Chen, Shu-Miao Zhang, Min Jia, Guo-Xu Zheng, Jian-Ming Pei, Li-Hua Chen

**Affiliations:** 1 Department of Physiology and Pathophysiology, National Key Discipline of Cell Biology, School of Basic Medicine, Fourth Military Medical University, No.169, West Changle Road, Xi'an, 710032, China.; 2 Department of Immunology, School of Basic Medicine, Fourth Military Medical University, No.169, West Changle Road, Xi'an, 710032, China.; 3 Department of Cardiology, Xijing Hospital, Fourth Military Medical University, No.169, West Changle Road, Xi'an, 710032, China.; 4 Faculty of Medicine, Northwest University, No.229, Taibai Bei Road, Xi'an, 710069, China

**Keywords:** macrophage, myocardial infarction, healing, inflammation, polarization

## Abstract

Macrophages are essential for wound repair after myocardial infarction (MI). CD226, a member of immunoglobulin superfamily, is expressed on inflammatory monocytes, however, the role of CD226 in infarct healing and the effect of CD226 on macrophage remain unknown.

**Methods:** Wild type and CD226 knockout (CD226 KO) mice were subjected to permanent coronary ligation. CD226 expression, cardiac function and ventricular remodeling were evaluated. Profile of macrophages, myofibroblasts, angiogenesis and monocytes mobilization were determined.

**Results:** CD226 expression increased in the infarcted heart, with a peak on day 7 after MI. CD226 KO attenuated infarct expansion and improved infarct healing after MI. CD226 deletion resulted in increased F4/80^+^ CD206^+^ M2 macrophages and diminished Mac-3^+^ iNOS^+^ M1 macrophages accumulation in the infarcted heart, as well as enrichment of α-smooth muscle actin positive myofibroblasts and Ki67^+^ CD31^+^ endothelial cells, leading to increased reparative collagen deposition and angiogenesis. Furthermore, CD226 deletion restrained inflammatory monocytes mobilization, as revealed by enhanced retention of Ly6C^hi^ monocytes in the spleen associated with a decrease of Ly6C^hi^ monocytes in the peripheral blood, whereas local proliferation of macrophage in the ischemic heart was not affected by CD226 deficiency. *In vitro* studies using bone marrow-derived macrophages showed that CD226 deletion potentiated M2 polarization and suppressed M1 polarization.

**Conclusion**: CD226 expression is dramatically increased in the infarcted heart, and CD226 deletion improves post-infarction healing and cardiac function by favoring macrophage polarization towards reparative phenotype. Thus, inhibition of CD226 may represent a novel therapeutic approach to improve wound healing and cardiac function after MI.

## Introduction

Myocardial infarction (MI) and subsequent heart failure are the leading causes of death worldwide. MI results in progressive loss of viable myocardium and adverse remodeling of the infarcted left ventricle (LV), leading to chamber dilatation, contractile dysfunction and eventually heart failure. Whereas proper and sufficient healing of ischemic injured myocardium prevents infarction expansion and preserves cardiac function with better long-term prognosis 1 MI triggers an inflammatory response which is mediated primarily by infiltrating immune cells including monocytes/macrophages, and accumulating evidence suggests that macrophages response following infarction is essential for cardiac healing 1-3. Although recognized as mediator of the prolonged and uncontrolled inflammation, contributing to myocardial injury and maladaptive remodeling after MI, macrophages are indispensably required for necrotic cell clearance, collagen deposition, and angiogenesis, thereby promoting infarct healing 4, 5. These diverse and seemingly conflicting observations may be attributable to macrophages heterogeneity.

Cardiac macrophages with distinct ontological origins represent a heterogeneous cell population of high plasticity that includes a spectrum of activation status ranging from pro-inflammatory (M1-type) to reparative (M2-type) phenotype, in response to cardiac microenvironment changes following myocardial injury like ischemia 1, 3, 6. The healing process after MI in murine involves the transition of pro-inflammatory to reparative macrophages, and excessive recruitment of inflammatory monocytes and activation of M1 macrophages retards infarct healing and worsens post-MI remodeling 1, 5. The existence of diverse macrophage subsets and their biphasic accumulation in ischemic heart represent challenge but also opportunities to elucidate the precise contributions of macrophage subsets to myocardial repair after MI. The expected and optimal healing is to ameliorate the detrimental effects of inflammatory macrophages, while sparing the beneficial healing roles of the reparative macrophages, which are depending on well-coordinated recruitment and activation of macrophages subpopulation 5, 7. The key molecules and related signaling pathways governing macrophage polarization toward pro-inflammatory or reparative phenotype, therefore, comprise novel potential targets of therapeutic intervention after MI.

CD226, also named as DNAX accessory molecule 1 (DNAM-1), is a 67-kDa type I membrane protein belonging to the immunoglobulin superfamily of receptors, and is constitutively expressed on the majority of T cells, natural killer (NK) cells, and monocytes 8, and the role of CD226 as an activating receptor on NK cells and T cells has been well studied 9, 10. However, very little is known about the role of CD226 in the diseased myocardium, and whether CD226 is involved in modulation of monocytes/ macrophags activation is poorly understood. A recent study indicated that CD226 is highly expressed on inflammatory monocytes, but not on patrolling monocytes 11. Our previous studies found that CD226 is highly expressed on activated human endothelial cells 12 and CD226 deletion improves cognitive functions and ameliorates anxiety-like behaviors in mice 13. Based on our work and that of others, we test the hypothesis that CD226 deletion may promote infarct healing by modulating macrophage polarization status, and could be exploited to improve ischemic wound repair and cardiac function. Here, we demonstrate that CD226 expression is remarkably increased in the infarcted heart after MI, and CD226 deletion favors macrophages activation status toward reparative phenotype in the ischemic heart, while restraining inflammatory monocytes mobilization from the spleen and peripheral blood, leading to increased reparative collagen deposition and angiogenesis, thereby contributing to infarct healing and preserved cardiac function.

## Methods

The detailed and expanded Methods section is available in the Supplemental Material.

### Mice

Mice with homozygous deletions of CD226 genes (*CD226^-/-^*) on a C57BL/6 background were kindly provided by Professor Marco Colonna (Washington University). C57BL/6 mice (8 weeks old) were purchased from Nanjing Biomedical Research Institute of Nanjing University (Nanjing, China). CD226^-/-^ (CD226 KO) mice were backcrossed to C57BL/6 strain, then propagated by CD226^+/-^ × CD226^+/-^ mating. Wild-type (WT) mice (*CD226^+/+^*) used as control in our experiments are the littermates of CD226 KO mice.

### Statistical analysis

Data were analyzed with GraphPad Prism-5 statistic software. All values are presented as the mean±SEM.

## Results

### CD226 expression is increased in the heart after MI

**Figure 1:**
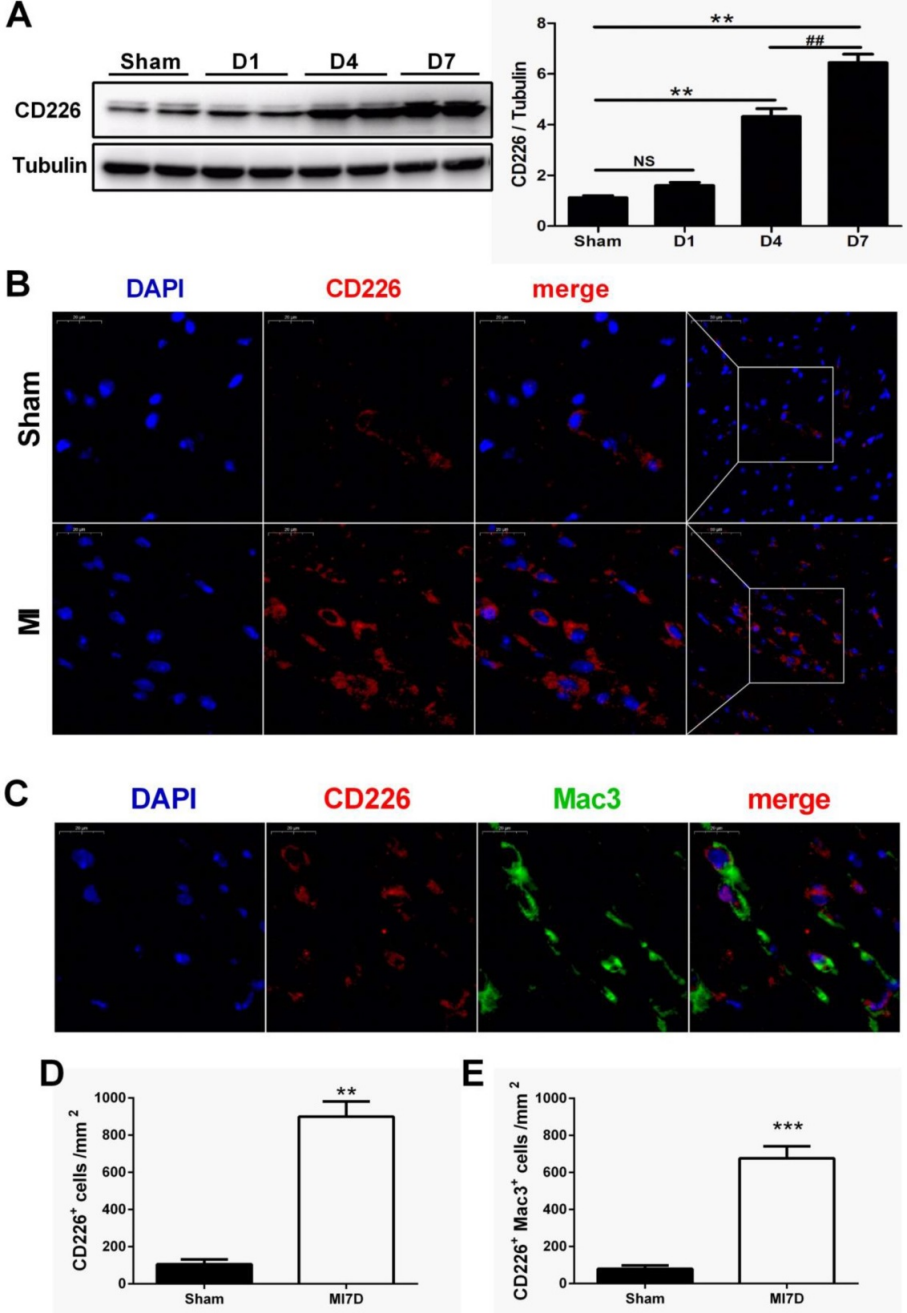
** CD226 expression is increased in the heart after myocardial infarction (MI) and CD226 expressing macrophages accumulate in the infarcted heart. A.** Representative immunoblots and summary data showing CD226 expression in infarcted heart tissue at different time points after MI. NS indicates no significance. ** *P* < 0.01 vs. sham and ^##^ *P* < 0.01 versus MI day 4, n=6 per group. **B.** Representative images of immunofluorescence staining for CD226 in the infarcted heart of wild type (WT) mice 7 days after MI or sham surgery. Scale bars as indicated (left 20 μm, right 50 μm). **C-E.** Immunofluorescent costaining for CD226 (red) and Mac-3 (green) in the infarcted heart of WT and CD226 KO mice (**C**), and quantitative analysis of CD226^+^ cells (**D**) and CD226^+^ Mac3^+^ macrophages (**E**) in the infarcted heart at day 7 after MI. DAPI was used for nuclear staining (blue). Scale bar =20 μm. n=6 mice per group, ** *P*<0.01, *** *P*<0.001.

To explore whether CD226 is expressed in the heart after MI and to define its cellular sources, we subjected wild-type (WT) C57BL/6N mice to permanent ligation of coronary left anterior descending (LAD) artery. CD226 expression was not altered one day after MI, but dramatically increased in the infarcted heart at day 4, and peaked at day 7 after MI when compared with the sham group (Figure 1A and Figure S1A). Immunofluorescence analyses also showed a marked increase in the expression of CD226 in the infarct and border area 7 days after MI compared to the sham group (Figure 1B and Figure S1B). Furthermore, dual immunofluorescence results revealed that CD226 and Mac-3 co-localization was commonly observed in the infarct and border zone, and CD226^+^Mac3^+^ macrophages accounted for around 74% of the CD226-expressing cells in the infarcted heart at day 7 after MI (Figure 1C-E and Figure S1C).

### CD226 deletion attenuates progressive ventricular dilation and ameliorates cardiac function after MI

**Figure 2:**
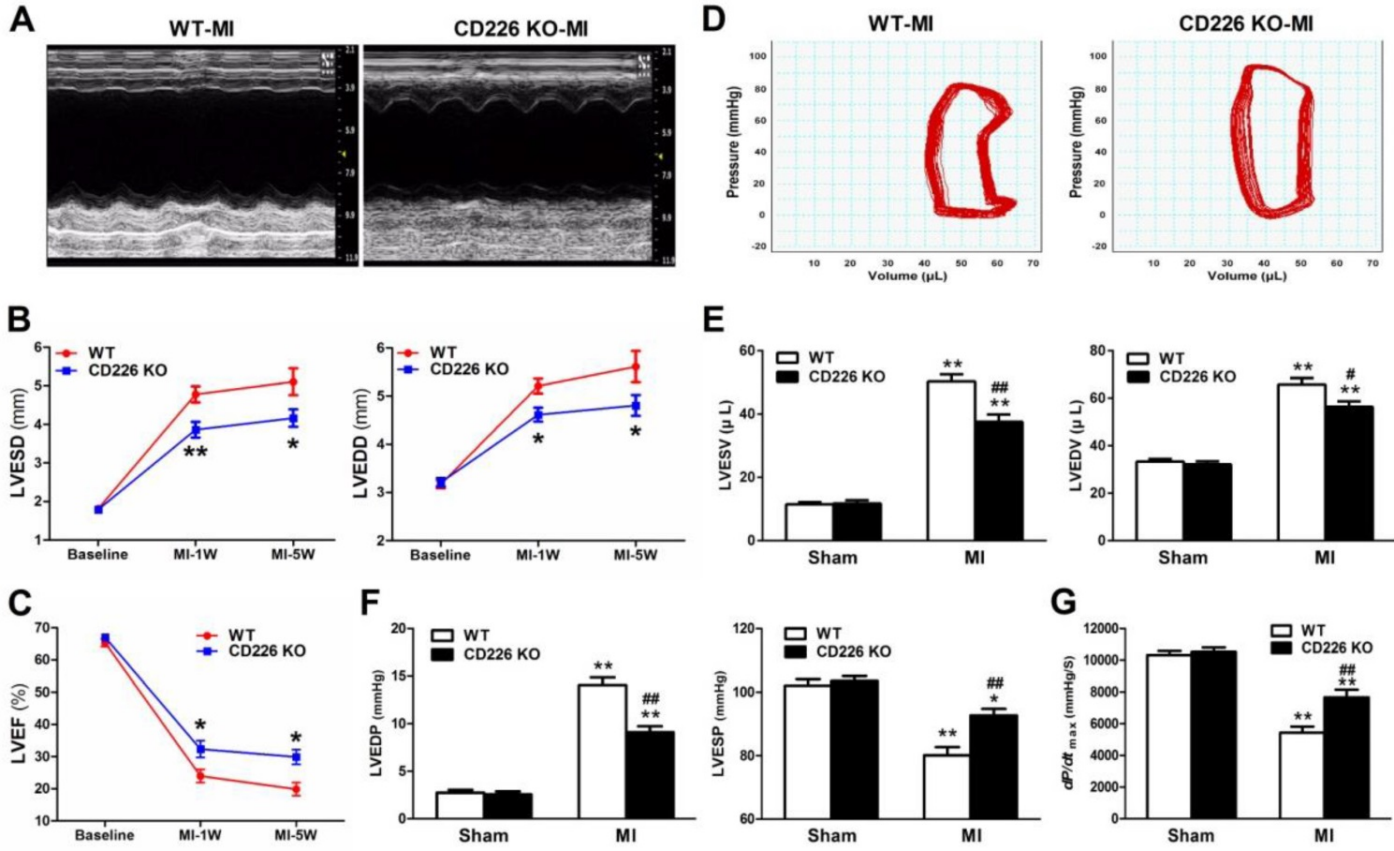
** CD226 deletion ameliorates cardiac dysfunction after MI. A.** Representative M-mode echocardiograms of infarcted hearts from WT and CD226 KO mice on day 7 after MI. **B-C**. Echocardiographic analysis of left ventricle end-systolic diameter (LVESD), LV end-diastolic diameter (LVEDD) and LV ejection fraction (LVEF) at pre-MI (baseline) and on 1 week and 5 weeks after MI. n=10 per group, * *P*<0.05 and ** *P*<0.01 vs. WT at corresponding time point. **D.** Representative LV pressure-volume loops of infarcted hearts from WT and CD226 KO mice 5 weeks after MI. **E-G.** LV end-systolic volume (LVESV), LV end-diastolic volume (LVEDV), LV end-systolic pressure (LVESP), LV end-diastolic pressure (LVEDP) and maximum of *dP/dt* were acquired from WT and CD226 KO mice at 5-week end point after MI or sham operation using Millar catheterization. n=8 per MI group, n=5 per sham group. * *P*<0.05, ** *P*<0.01 vs corresponding Sham, and ^#^ *P* < 0.01, ^##^ *P* < 0.01 vs WT MI. Data are expressed as mean±SEM.

To determine the effect of CD226 deletion on cardiac function and myocardial remodeling, WT and CD226 KO mice were subjected to MI. LAD coronary ligation induces transmural ischemia as evidenced by rapid ST-segment elevation on the ECG within 30 seconds after ligation (Figure S2). Baseline (one day before LAD ligation) echocardiograms showed that WT and CD226 KO hearts exhibited comparable ventricular function. As early as 1 week after MI, both WT and CD226 KO hearts developed decreased LV ejection fraction (LVEF) and increased LV chamber dimension, suggesting cardiac dysfunction and LV dilatation following MI. However, LV dilatation was ameliorated in CD226 KO mice compared with WT mice, as evidenced by reduced LV end-systolic dimension (LVESD) and LV end-diastolic dimension (LVEDD) (Figure 2A-2B). This was associated with more preserved cardiac function in CD226 KO mice, as reflected by greater LVEF (Figure 2C). The progressive ventricular dilation and cardiac dysfunction observed by serial echocardiography in WT mice was ameliorated in CD226 KO animals throughout the duration of study (Figure 2B-2C).

We further measured hemodynamic parameters using an invasive catheter 5 weeks after MI. MI resulted in dramatically rightward shift of the pressure-volume curve in WT mice, and this was attenuated in CD226 KO mice (Figure 2D and Figure S3). LV end-systolic volume (LVESV), LV end-diastolic volume (LVEDV) and LV end-diastolic pressure (LVEDP) were significantly decreased, whereas, LV end-systolic pressure (LVESP) was much more preserved in CD226 KO mice in comparison to WT mice (Figure 2E-2F and Figure S4A). Moreover, CD226 KO mice showed increased *dp/dt*_max_ values than WT mice, suggesting improved cardiac contraction capacity (Figure 2G and Figure S4B). Together, these results demonstrate that CD226 deletion attenuates progressive ventricular dilation and restores cardiac function after MI.

### CD226 deletion prevents infarct expansion and attenuates cardiac remodeling after MI

**Figure 3:**
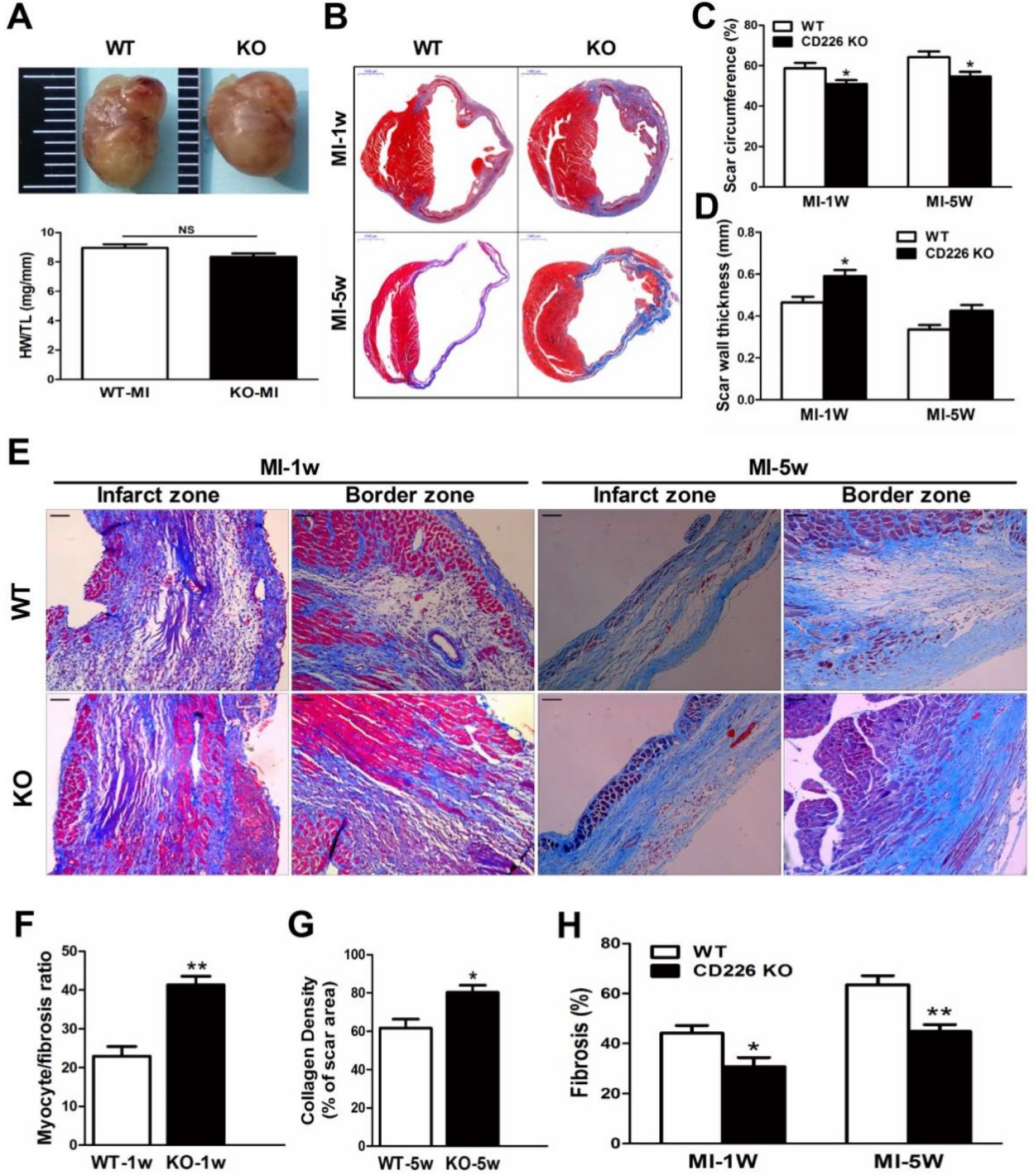
** CD226 deletion prevents infarct expansion and ameliorates cardiac remodeling after MI. A.** Gross observation of infarcted hearts (top) and the ratio of heart weight to tibia length (HW/TL) (bottom). **B.** Representative images of Masson trichrome staining of heart cross sections of WT and CD226 KO mice at 1 week and 5 weeks after MI. Scale bar =1 mm. **C-D.** Infarct scar circumference (**C**) was expressed as a percentage of LV including the septum, and wall thickness of infarct scar (**D**) at papillary muscle level was measured 1week and 5 weeks after MI. n=6 in each group at each time point, * *P*<0.05 vs. corresponding WT. **E.** CD226 KO mice exhibited more preserved myocardium (1 week post-MI) and increased collagen deposition (5 weeks post-MI) in the infarct zone, and decreased interstitial fibrosis in the border zone. Scale bar =50 μm. **F-H**. Ratio of myocyte to fibrosis 1 week after MI (**F**) and collagen density 5 weeks after MI (**G**) in the infarct zone, and collagen volume fraction in the border zone (**H**) were quantitatively estimated in 5 randomly chosen high-power fields (×200) in each section. Results obtained from the same heart were averaged and counted as n=1. n = 6 in each group at each time point, * *P*<0.05 and ** *P*<0.01 vs. corresponding WT. Data are expressed as mean±SEM.

The heart size and heart weight/tibia length tended to be lower in CD226 KO mice 1 week after MI in comparison to WT mice (Figure 3A). Histological analysis revealed that there was no morphological difference in the sham-operated hearts between WT and CD226 KO mice (Figure S5). However, infarcted hearts of WT mice were more dilated and the infarction pattern spread far beyond the LV anterior wall in comparison to infarcted hearts of CD226 KO mice throughout the study duration (Figure 3B and Figure S5). Scar tissue percent circumference was significantly decreased and scar wall thinning was retarded in CD226 KO hearts at both 1 week and 5 weeks after MI (Figure 3C-3D). Although there was not statistically different of the post-MI survival rate between the two groups, (The percent survival at 35 days: CD226 KO, 70.3% vs. WT, 59.7%, P=0.446, n=30-34. Figure S6A), the LV tissues was more preserved and infarct wall was thicker in CD226 KO hearts among mice died 5 days after MI (Figure S6B). CD226 KO mice exhibited more surviving myocardium in the infarct scar and significantly increased ratio of myocytes to fibrotic tissues in the infarct zone 1 week after MI (Figure 3E-3F and Figure S7), and increased density of collagen deposition in the infarct region 5 weeks after MI (Figure 3E and 3G, Figure S9). In addition, both masson's trichrome and picrosirius red staining revealed a disorganized scar formation with a predominance of loosely and fragmented collagen fibers in WT mice, in contrast to a well-organized scar with well-aligned collagen fibers in CD226 KO mice (Figure 3E, and Figure S7-9), suggesting reparative collagen deposition and improved healing of the infarcted myocardium in CD226 KO mice. Myocardial interstitial fibrosis in the infarct border zone was significantly attenuated in CD226 KO mice compared with WT mice (Figures 3E and 3H, and Figure S8-9). These results suggest that CD226 deletion inhibits infarct expansion and exerts a cardio-protective effect on post-MI remodeling and healing.

### Increased accumulation of F4/80^+^ CD206^+^ M2-like macrophages and less Mac-3^+^ iNOS^+^ M1-like macrophages in the ischemic heart of CD226 KO mice

**Figure 4:**
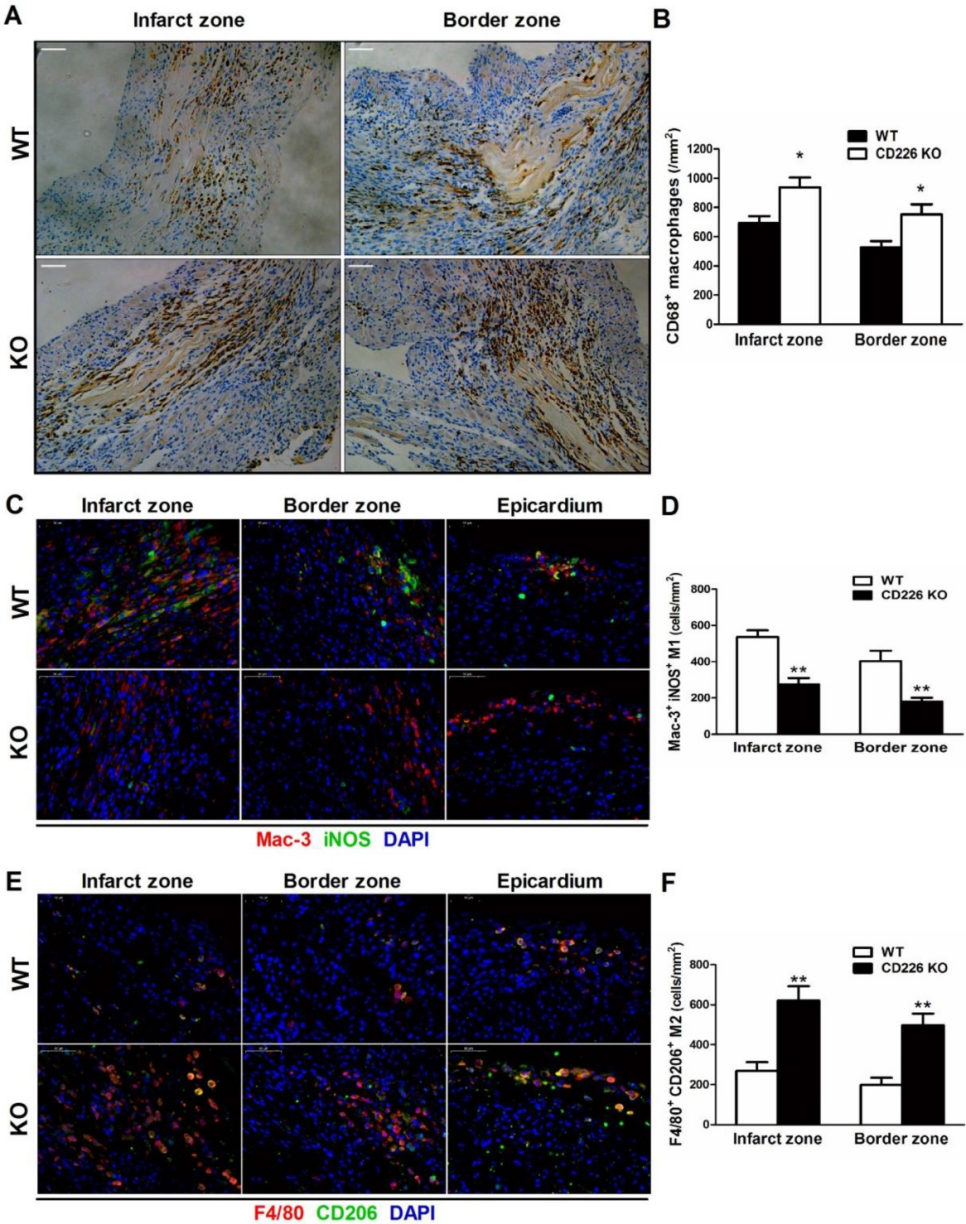
** CD226 deletion results in increased accumulation of F4/80^+^ CD206^+^ macrophages and diminished Mac-3^+^ iNOS^+^ macrophages in the ischemic heart after MI. A** and** B.** Immunohistochemical staining for CD68 (**A**) and quantitative analysis of CD68^+^ macrophages (**B**) in the infarct zone and border zone of WT and CD226 KO mice at day 7 after MI. Scale bar=50 μm. n=5 mice per group, * *P*<0.05 vs. WT. **C** and** D.** Representative immunofluorescence photographs of Mac-3^+^ (red) and iNOS^+^ (green) macrophages and quantitative analysis of Mac-3^+^ iNOS^+^ M1 macrophages in the infarct, border zone and epicardium area of WT and CD226 KO mice at day 7 after MI. DAPI was used for nuclear staining (blue). Scale bar =50 μm. n=5 mice per group, ** *P*<0.01 vs. WT. **E** and** F**. Representative immunofluorescence photographs of F4/80^+^ (red) and CD206^+^ (green) macrophages and quantitative analysis of F4/80^+^ CD206^+^ M2 macrophages in the infarct, border zone and epicardium area of WT and CD226 KO mice at day 7 after MI. Scale bar =50 μm. n=5 mice per group, ** *P*<0.01 vs corresponding WT.

MI induced a remarkable increase in the infiltration of CD68^+^ macrophages in the infarct zone, and the border area (albeit to a lesser extent) 7 days after MI, whereas, few CD68^+^ macrophages were observed in the remote area of infarct, as well as in the sham group. The number of CD68^+^ differentiated macrophages within the infarct and border zone was significantly higher in CD226 KO mice compared with WT mice (Figure 4A-4B, and Figure S10). Equivalent data were obtained with staining for Mac-3, another marker for macrophage. However, Ly6G^+^ neutrophils were rarely observed in the ischemic heart 7 days after MI (Figure S11). Notably, increased accumulation of CD68^+^ macrophages corresponded to reduced interstitial fibrosis and well-aligned collagen fibers in the infarct border zone, as observed in CD226 KO mice in contrast to WT mice (Figure S12). Another observation deserve attention is that there was less CD68^+^ macrophages infiltration in the infarcted hearts of mice died within one week after MI, and this trend was more obvious in WT mice than CD226 KO mice (Figure S13).

We further explored whether CD226 deletion affect macrophages phenotype within the infarct healing microenvironment. There were enhanced accumulation of F4/80^+^ CD206^+^ M2 macrophages and diminished recruitment of Mac-3^+^ iNOS^+^ M1 macrophages in the infarcted heart of CD226 KO mice 7 days after MI compared with WT mice (Figure 4C-4F and Figure S14-15). Intriguingly, we observed that many F4/80^+^ CD206^+^ M2-like macrophages and Mac-3^+^ iNOS^+^ M1-like macrophages localized on the epicardial surface and in the subepicardial space in addition to the infarct area (Figure S16), which corroborate previous report that epicardial activation is involved in promoting leukocyte recruitment and inflammatory responses 14. In line with the date obtained in the infarct area, there were more M2-like macrophages and less M1-like macrophages in the perivascular and epicardial area of CD226 KO mice in comparison to WT mice (Figure S16-17).

### CD226 deletion restrains monocytes mobilization from the blood and spleen, without affecting proliferation of cardiac macrophages

To determine whether alterations of macrophage activation status (increased accumulation of M2-type macrophages and reduced number of M1-type macrophages) in the infarcted hearts of CD226 KO mice result from increased proliferation of local macrophages or decreased infiltration of inflammatory monocytes 6, 15, we further explored proliferation of local macrophages in the ischemic heart. Proliferating macrophage was identified as dual positive of F4/80 and Ki67 by immunofluorescence analysis, and the number of F4/80^+^ Ki67^+^ macrophages in the infarcted heart was comparable between WT and CD226 KO mice, suggesting that proliferation of local macrophages was not affected by CD226 deletion (Figure S18).

**Figure 5:**
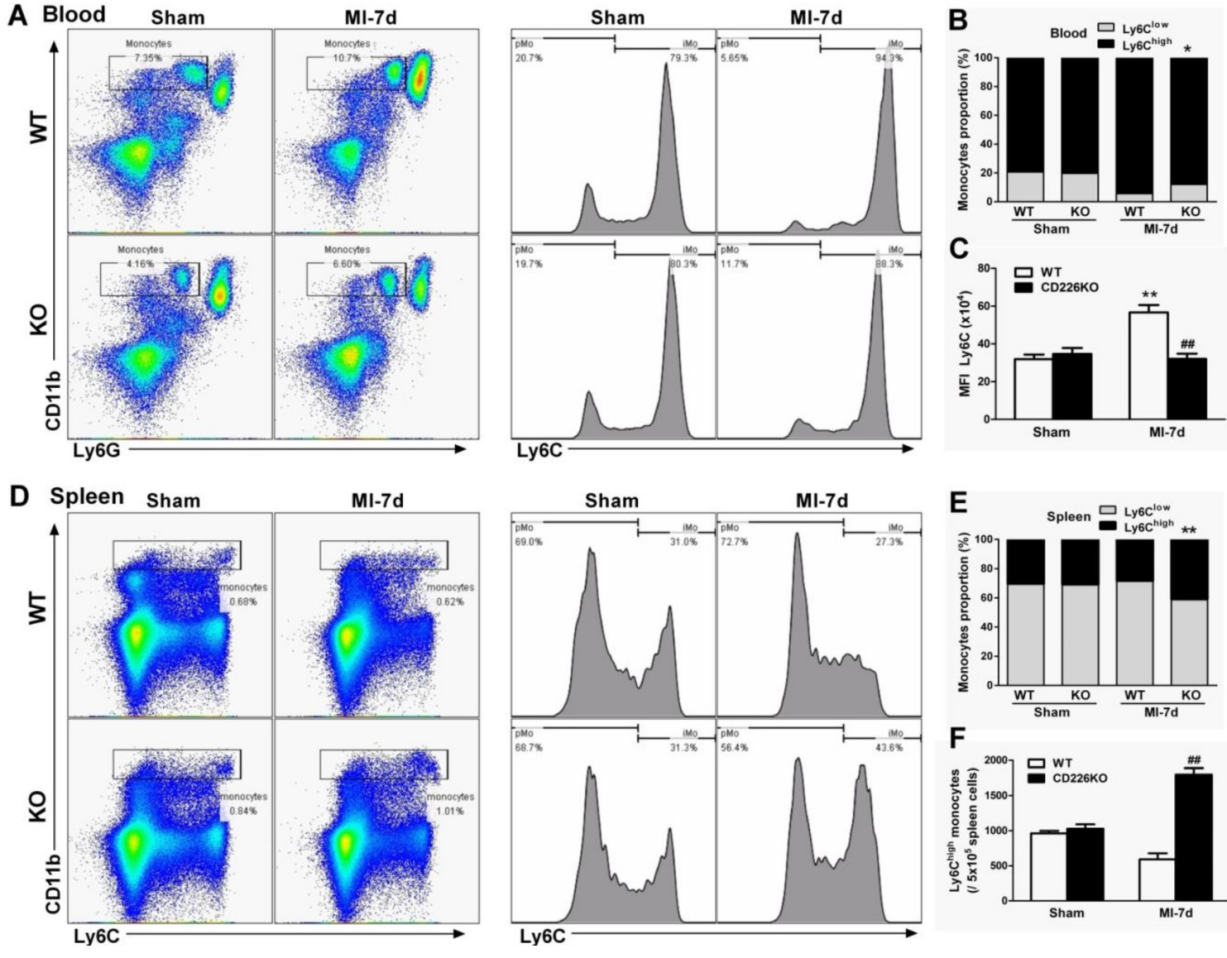
** CD226 depletion restrains monocytes mobilization from the spleen and peripheral blood. A.** Representative flow cytometric dot plots and histograms show Ly6C^high^ and Ly6C^low^ monocytes in the peripheral blood of WT and CD226 KO mice 7 days after MI or sham surgery. Numbers indicate the percentage of Ly6C^high^ or Ly6C^low^ cells within the cell population analyzed. **B.** Ly6C^high^ and Ly6C^low^ monocytes proportion in the blood. * *P* < 0.05 vs. WT-MI. **C.** Mean fluorescent intensity (MFI) of Ly6C on peripheral blood Ly6C^high^ monocytes at day 7 after MI. n = 6 mice per group. Data are represented as mean values ± SEM. ** *P* < 0.01 vs. WT-Sham, ^##^ *P* < 0.01 vs. WT-MI. **D**. Representative flow cytometric dot plots and histograms show Ly6C^high^ and Ly6C^low^ monocytes in the spleen of WT and CD226 KO mice. Numbers indicate the percentage of Ly6C^high^ or Ly6C^low^ cells within the cell population analyzed. **E.** Ly6C^high^ and Ly6C^low^ monocytes proportion in the spleen 7 days after MI or sham surgery. ** *P* < 0.01 vs. WT-MI. **F.** Number of Ly6C^high^ monocytes in the spleen at day 7 after MI. n = 6 mice per group. Mean values ± SEM are represented. ^##^ *P* < 0.01 vs WT-MI.

In response to cardiac injury like MI, marked expansion of macrophages population in the stressed heart originate from the spleen via bloodstream 16 in addition to *in situ* proliferation of resident macrophages 15. We investigated whether CD226 deletion affect mobilization and recruitment of monocytes from peripheral blood and spleen. Flow cytometric analysis demonstrated that MI resulted in an increase in the number of Ly6C^high^ monocytes and increased proportion of Ly6C^high^ monocytes relative to Ly6C^low^ monocytes in the peripheral blood of WT mice, and this trend was blunted in CD226 KO mice (Figure 5A-5B). More importantly, WT blood Ly6C^high^ monocytes showed much higher mean fluorescence intensity (MFI) values of Ly6C than Ly6C^high^ monocytes of CD226 KO mice after MI (Figure 5C). MI resulted in a decrease in the number of Ly6C^high^ monocytes and decreased proportion of Ly6C^high^ monocytes in the spleen. However, CD226 KO mice exhibited retention of Ly6C^high^ monocytes in the spleen following MI, as evidenced by an increase in the number and proportion of Ly6C^high^ monocytes in the spleen (Figure 5D-5F), suggesting that CD226 deletion restrains mobilization of Ly6C^high^ pro-inflammatory monocytes from the spleen via peripheral blood after MI.

### CD226 deletion facilitates myofibroblasts accumulation and angiogenesis in the infarcted heart

**Figure 6:**
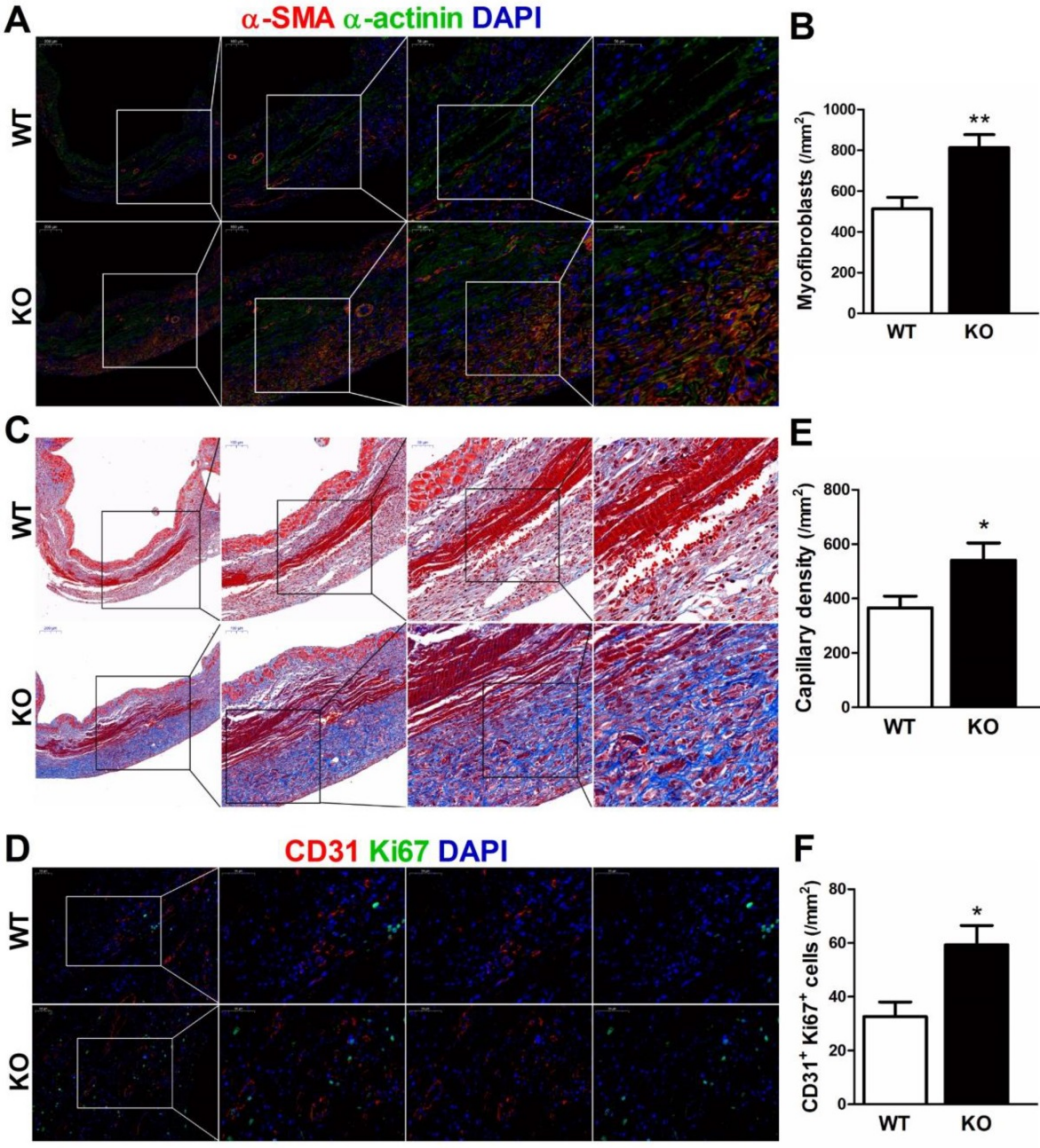
** CD226 deletion promotes myofibroblasts accumulation and angiogenesis in the infarcted heart after MI. A-B.** Representative staining for α-smooth muscle actin (α-SMA) and α-actinin, and quantitative analysis of α-SMA^+^ myofibroblasts in the infarcted heart 7 days after MI. CD226 KO mice exhibited an increase in the number of extravascular α-SMA^+^ spindle-shaped myofibroblasts in the infarct area. **C.** Masson trichrome staining of serial heart sections revealed higher density of collagen deposition with well-aligned collagen fibers in the infarct zone of CD226 KO mice. **D**. Representative immunofluorescence staining of CD31^+^ capillaries and Ki67^+^ CD31^+^ proliferating endothelial cells in the infarct border zone at day 7 after MI. **E-F.** Quantitative analysis of CD31^+^ capillaries and Ki67^+^ CD31^+^ proliferating endothelial cells in the infarct border zone 7 days after MI. n=4 mice per group, * *P*<0.05 and ** *P*<0.01 vs. WT. Data are expressed as mean±SEM.

During the proliferative phase of infarct healing, cardiac fibroblasts mainly of intracardiac origin, migrate into the infarct area and differentiate into myofibroblasts, which are crucial for the prevention of infarct expansion and LV rupture 17. Accordingly, we examined whether infarct healing microenvironment dominated by M2 macrophages lead to reparative properties of fibroblasts in CD226 KO mice. As showed by serial sections staining analyses, CD226 KO mice exhibited significantly increased numbers of extravascular α-SMA-positive spindle-shaped myofibroblasts and higher density of collagen deposition with well-aligned collagen fibers in the infarcted hearts 7 days after MI (Figure 6A-6C, and Figure S19).

Next, we investigated whether CD226 deletion and subsequent increased M2 macrophages would affect angiogenesis in the ischemic heart. Indeed, we observed increased number of CD31-positive capillaries and Ki67^+^ CD31^+^ proliferating endothelial cells in the infarct border zone of CD226 KO mice 7 days after MI in comparison to WT mice (Figure 6D-6F). However, endothelial cell proliferation and capillary density in the noninfarcted remote region and the sham-operated heart were comparable between WT and CD226 KO mice. Moreover, arhgap31 was identified as a differentially expressed protein (detail in Figure 8), and a ~50% increase in the expression of arhgap31 was detected in the infarcted heart of CD226 KO mice relative to WT mice, which is in agreement with previous finding that arhgap31 is essential to angiogenesis 18. These findings suggest that CD226 deletion results in enrichment of α-SMA^+^ myofibroblasts, and subsequent reparative collagen deposition and angiogenesis in the infarcted heart.

### CD226 deletion potentiates macrophage polarization toward M2 phenotype and suppresses M1 polarization

**Figure 7:**
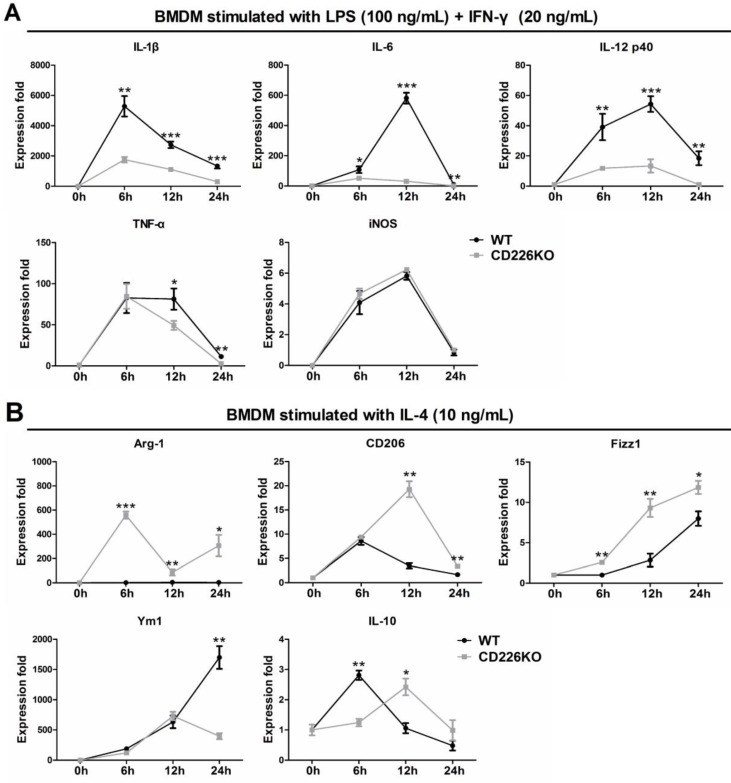
** CD226 deletion suppresses M1 polarization and potentiates M2 polarization in bone marrow derived macrophages (BMDM).** BMDM were isolated from WT and CD226 KO mice. **A.** Time course changes of M1 polarization marker genes in BMDM, upon stimulation with LPS (100 ng/mL) + IFN-γ (20 ng/mL) for 6, 12 and 24 hours as indicated. CD226 deletion suppressed M1 polarization as evidenced by decreased mRNA levels of IL-1β, IL-6, IL-12p40 and TNF-α. n = 4 independent experiments. * *P*<0.05, ** *P*<0.01, *** *P*<0.01 vs. WT. **B.** Profile of M2 polarization marker genes in BMDM in the presence of IL-4 (10 ng/mL) for 6, 12 and 24 hours as indicated. CD226 deletion potentiated M2 macrophage polarization as evidenced by increased mRNA levels of Arg1, CD206, Fizz1, Ym1, IL-10. n = 4 independent experiments. * *P*<0.05, ** *P*<0.01, *** *P*<0.01 vs. WT.

To confirm the *in vivo* findings and explore the underlying mechanism, we investigated the effects of CD226 deletion on macrophage polarization, using bone marrow (BM)-derived macrophages isolated from WT and CD226 KO mice. BMDM cells were differentiated into macrophages and stimulated with IFN-γ (20 ng/mL) + LPS (100 ng/mL) or IL-4 (10 ng/mL) for different times as indicated, and mRNA level of typical M1 and M2 macrophage marker genes were measured and profiled. Following treatment with LPS + IFN-γ, mRNA levels of M1 marker genes including IL-1β, IL-6, IL-12p40, TNF-α, iNOS were strongly up-regulated in WT macrophages, whereas mRNA expression of IL-1β, IL-6, IL-12p40 were significantly suppressed in CD226 deficiency macrophages, with the exception of iNOS (Figure 7A). IL-4 stimulation induced undetectable expression of Arg1, and moderate increase in the mRNA levels of CD206, Fizz1 and IL-10 in WT macrophages, whereas mRNA expression of these M2 marker genes were strikingly heightened in CD226 deficiency macrophages in the presence of IL-4 stimulation (Figure 7B). Thus, CD226 was identified as a novel molecule governing macrophage polarization balance, and CD226 deletion favors M2 polarization and suppresses M1 polarization.

Mechanisms by which CD226 regulates macrophage polarization in the infarct is a key question we try to answer. Next, we detected the effect of CD226 knockout on NF-κB p65 and STAT6 phosphorylation in BMDMs and found that CD226 knockout may increase a little higher STAT6 phosphorylation in BMDMs (although *P*>0.05 corresponding WT, **Figure S20A-B**). Then we detected the effect of CD226 knockout on NF-κB p65 and STAT6 phosphorylation in peritoneal macrophages and found that CD226 knockout increased STAT6 phosphorylation in peritoneal macrophages (**Figure S21A-B**).

### Quantitative proteomic analysis of differentially expressed proteins in the infarcted heart

**Figure 8:**
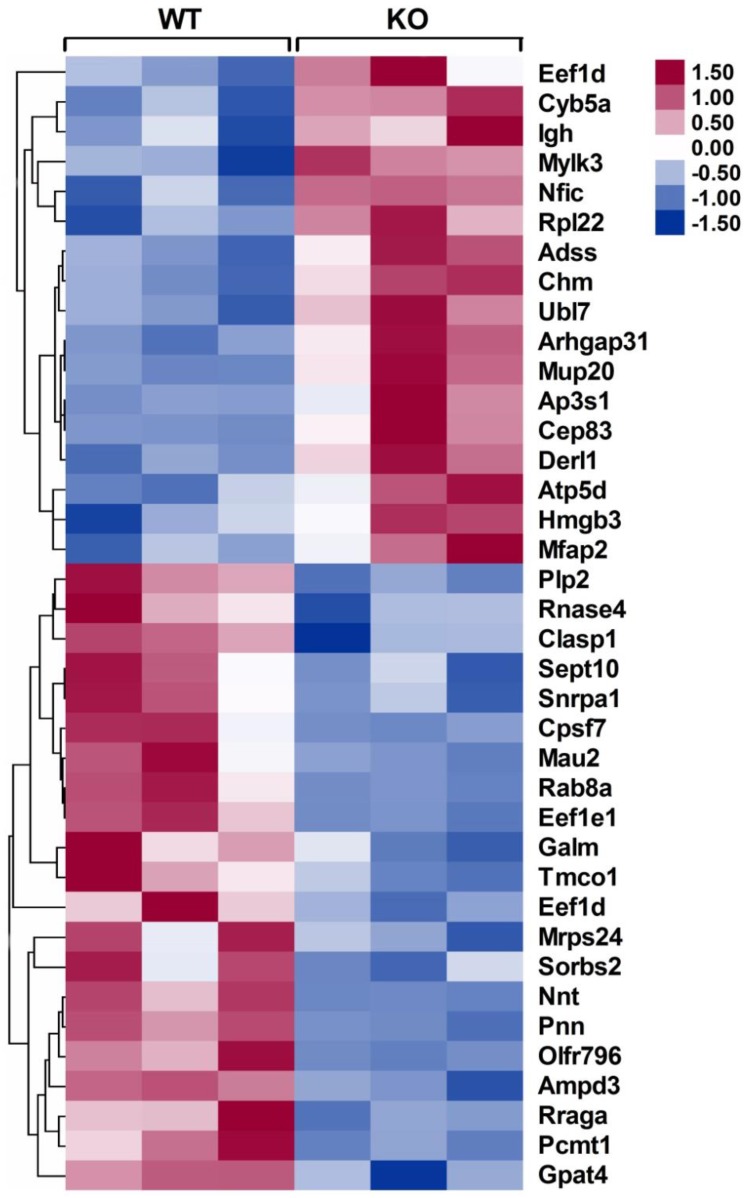
** Heat maps of differentially expressed proteins in the infarcted hearts of WT and CD226 KO mice.** Inserted, scale of -fold change. Following mass spectrometry, we set an arbitrary threshold of 1.2-fold to declare differences, 17 proteins were up-regulated and 21 proteins were down-regulated in the infarcted hearts of CD226 KO mice compared with WT mice. n= 3 per group, and each sample was the mixture of two infarcted hearts of the same group.

To identify the differentially expressed proteins that may be responsible for improving infarct healing and cardiac function, an independent and nonbiased proteomics analysis, isobaric tags for relative and absolute quantification (iTRAQ), was performed using infarcted hearts of WT and CD226 KO mice, and a 1.2-fold change was chosen as the threshold for significant changes in protein expression. Using these criteria, the expression levels of 38 proteins were changed in the infarcted hearts of CD226 KO mice compared with WT group (Figure 8 and Table S1-2). Of these, 17 proteins including cardiac myosin light-chain kinase (cMLCK, encoded by *Mylk3*) were up-regulated (Table S1), and 21 proteins including mitochondrial nicotinamide nucleotide transhydrogenase (Nnt) and AMP deaminase 3 (AMPD3) were down-regulated (Table S2).

Among the up-regulated proteins, cMLCK has been demonstrated to be critical for adaption to cardiac stress, and mice with cMLCK overexpression were protected from pressure overload, whereas cMLCK KO mice were predisposed to severe heart failure 19. In line with this finding, the increased expression of cMLCK in the infarcted heart of CD226 KO mice (ratio of KO/WT is 1.51, *P* = 0.007) may contribute, at least partially, to the preserved cardiac function. Among the down-regulated proteins, Nnt normally regenerates NADPH from NADH, but reverses Nnt depletes NADPH and induces mitochondrial oxidative stress and necrosis in the presence of pressure overload, whereas Nnt defective mice were protected from oxidative stress and heart failure 20. Nnt expression was significantly decreased in infarcted heart of CD226 KO mice (ratio of KO/WT is 0.287, *P* = 0.002), which may contribute to reduced infarct expanssion and improved cardiac function. Moreover, AMPD3 is the heart type AMP deaminase, catalysing the conversion of AMP to IMP, and increased cardiac AMPD3 expression and activity is associated with ATP depletion and diastolic dysfunction in pressure overloaded rat hearts 21. Consistent with this finding, the decreased level of AMPD3 in the ischemic heart of CD226 KO mice (ratio of KO/WT is 0.811, *P* = 0.001) may contribute to improved cardiac function. However, the precise role of these differentially expressed proteins in ischemic injury and infarct healing needs to be investigated in the future study.

## Discussion

Here, we demonstrate for the first time that CD226 expression increased in the infarcted heart following MI, and CD226 deletion modulates macrophages activation status toward a reparative profile in the ischemic heart, while restraining inflammatory monocytes mobilization from the spleen and peripheral blood, leading to reparative collagen deposition and angiogenesis, contributing to infarct healing. Moreover, CD226 is identified as a novel molecule governing macrophage polarization balance, and CD226 deletion favors M2 polarization and suppresses M1 polarization.

Very little is known about the role of CD226 in the diseased myocardium. CD226, also named as DNAM-1, was originally discovered as an adhesion molecule that is constitutively expressed on the majority of peripheral blood T lymphocytes and NK cells and plays an important role in the T cell and NK cell mediated cytotoxicity and immunity to a variety of tumors 8-10. Later, CD226 was also found to promote inflammation-driven dermal fibrosis in a mouse model of systemic sclerosis 22 and contribute to development of acute graft-versus-host disease (GVHD) in mice 23. Our recent study showed that CD226 deficiency improves cognitive functions and ameliorates anxiety-like behaviors in mice 13. Another study describes the high expression of CD226 on inflammatory monocytes, but not on patrolling monocytes 11. Here, we found that CD226 expression increased gradually in the infarcted heart, peaked at day 7 after MI, and a large number of CD226-expressing cells are macrophages. However, whether CD226 is involved in cardiac macrophage phenotype modulation and infarct repair remains to be determined.

Monocytes and macrophages play a crucial role in the inflammation and injury repair after MI^24^,^25^. On the one hand, excessive infiltration and activation of monocytes/macrophages is associated with augmented inflammation and myocardium injury, leading to infarct expansion and adverse LV remodeling in mice 5, 7, On the other hand, macrophage depletion in infarcted hearts impairs necrotic cell clearance, collagen deposition, and angiogenesis, predisposing to cardiac rupture and heart failure, suggesting macrophage is essential and indispensible for infarct healing 4. These diverse and seemingly conflicting observations may be attributed to macrophages heterogeneity. Seminal studies by Nahrendorf and coworkers showed that post-infarction repair process consists of biphasic accumulation of 2 distinct subsets of monocytes/macrophages. The Ly6C^high^ proinflammatory monocytes dominate the early phase (1 to 3 days) and exhibit phagocytic, proteolytic, and inflammatory properties, and Ly6C^low^ reparative monocytes dominate the late phase (4 to 7 days) and promote angiogenesis and resolution of inflammation 4, 7, 16. Thereafter, the phenotypic plasticity and functional heterogeneity of macrophages has gained increasingly attention in the pathogenesis of cardiovascular disease, and accumulating evidence have suggested that indiscriminate or broad immunosuppression is detrimental for infarct healing because many macrophage actions, M2 reparative macrophages in particular, are essential for efficient repair program and reconstitution of tissue integrity 6, 26-29.

The existence of diverse macrophages subsets and their biphasic accumulation in ischemic myocardium represents challenge, but also offer opportunities to dissect the precise role of a certain subset of macrophages in myocardial injury and infarct repair after MI. The expected and optimal healing is to ameliorate the detrimental effects of inflammatory macrophages, while sparing the beneficial healing roles of reparative macrophages. With the understanding of cardiac macrophages subsets constitution 6, and the recent insights into molecular mechanisms underlying phenotypic plasticity of macrophage 1, 3, it has become feasible to inhibit infiltration of pro-inflammatory monocytes/ macrophages and/or modulate macrophage actions to optimize healing of injured tissues. One attractive option is to shift macrophage polarization towards M2-like phenotype, thereby promote inflammation resolution and infarct healing. Indeed, previous studies in ischemic heart indicated that *in vivo* silencing of interferon regulatory factor 5 (IRF5), a master transcription factor governing macrophage polarization, suppressed M1 macrophage activation and supported infarct healing 30. Whereas deletion of Trib1, a Ser/Thr protein kinase essential to the differentiation of tissue M2-like macrophages, resulted in suppression of M2-like macrophages and impaired repair of the infarcted myocardium 28.

Our study uncovered the previously unknown role of CD226 in infarct healing by regulating macrophages phenotypes in the infarcted heart. CD226 deletion resulted in an increase of F4/80^+^ CD206^+^ M2 macrophages and a decline of Mac-3^+^ iNOS^+^ M1 macrophages in both infarct and border zone, suggesting accelerated transition of proinflammatory macrophages to reparative macrophages in ischemic myocardium. Although local proliferation of cardiac macrophages was not altered, CD226 deletion restrained inflammatory monocytes mobilization from the spleen and peripheral blood after MI. Moreover, CD226 KO mice exhibited enrichment of α-SMA^+^ myofibroblasts with high density of well-aligned collagen fibers in the infarct region, and an increase of proliferating Ki67^+^ CD31^+^ endothelial cells with associated angiogenesis in the infarct border zone. These findings suggest that CD226 deletion results in a reparative healing microenvironment dominated by M2-like macrophages, thereby attenuating LV dilation and ameliorating cardiac dysfunction after MI. Taken together, our work adds to and extends recent publications describing the role of macrophages in infarct repair 1, 3, 5, and further strengthens the notion that modulating macrophages in ischemic heart towards reparative phenotype is a promising therapeutics to promote inflammation resolution and infarct healing after MI.

Although CD226 is highly expressed on inflammatory monocytes 11, its precise role in modulation of monocytes/macrophages phenotype and function is poorly understood. Using BM-derived macrophages isolated from WT and CD226 KO mice, we identified CD226 as a novel modulator governing macrophages polarization balance, and CD226 deletion favors M2 polarization and suppresses M1 polarization. An interesting aspect of our study is the dual function of CD226 deletion in activating M2 genes while repressing M1 genes, which is in line with the paradigm that M1 and M2 genes are reciprocally regulated by the same transcription factor in macrophage polarization, as exemplified by the direct activation of M2 genes while repression of M1 genes by IRF5 31. More importantly, dual-modulation of macrophage polarization by CD226 deletion observed in our study may have broader implications in optimizing repair of injured tissues in a number of autoimmune and inflammatory diseases.

Our iTRAQ analysis identified a number of differentially expressed proteins in the infarcted heart. Of note, the up-regulated expression of cMLCK in infarcted heart may contribute to the preserved cardiac function in CD226 KO mice, which is consistent with previous study that cMLCK overexpressed mice were protected from pressure overload, whereas cMLCK KO mice were predisposed to severe heart failure 19. Among the down-regulated proteins, AMPD3 is the heart type AMP deaminase, catalysing the conversion of AMP to IMP, and increased AMPD3 expression and activity lead to ATP depletion and diastolic dysfunction after pressure overload in rat 21. Moreover, Nnt defective mice were protected from pressure overload induced heart failure and death 20. In agreement with these findings, the decreased expression of AMPD3 and Nnt in the infarcted heart may account, at least partly, for the attenuated LV remodeling and improved cardiac function in CD226 KO mice. However, the precise role of these differentially expressed proteins in myocardial ischemic injury and repair is not well appreciated, and its interaction with cardiac macrophages in the ischemic heart remains to be determined.

The limitation of the present study is using of CD226 global KO mice to determine the effects of CD226 deficiency on macrophages, and we can't rule out the cellular effects mediated by other CD226-expressing cells including T lymphocytes. The present data instigate our future study to dissect the specific effect of CD226 deletion on macrophages and T cells using myeloid cell specific CD226 KO mice and T cell specific CD226 KO mice, respectively, in the context of infarcted heart healing and remodeling.

In conclusion, the present work uncovers a previously unappreciated role of CD226 in infarct healing and cardiac remodeling by modulating macrophages polarization after MI. Inhibition of CD226 may represent a novel therapeutic approach to improve wound repair in the infarcted heart and other cardiovascular disease. Our data also reinforce the notion that the phenotypes of macrophages in the ischemic heart can be modulated to optimize infarct healing and adaptive remodeling.
