# Supplementary material for: CD226 deletion improves post-infarction healing via modulating macrophage polarization in mice
Source: Theranostics. 2020 Jan 20;10(5):2422–35. doi: 10.7150/thno.37106 (PMC7019150; doi:10.7150/thno.37106)
Supplement: Supplementary file 1 — Supplementary figures and tables. [file thnov10p2422s1.pdf]

CD226 deletion improves post-infarction healing via modulating macrophage polarization in mice

Supplemental Material

Figure S1

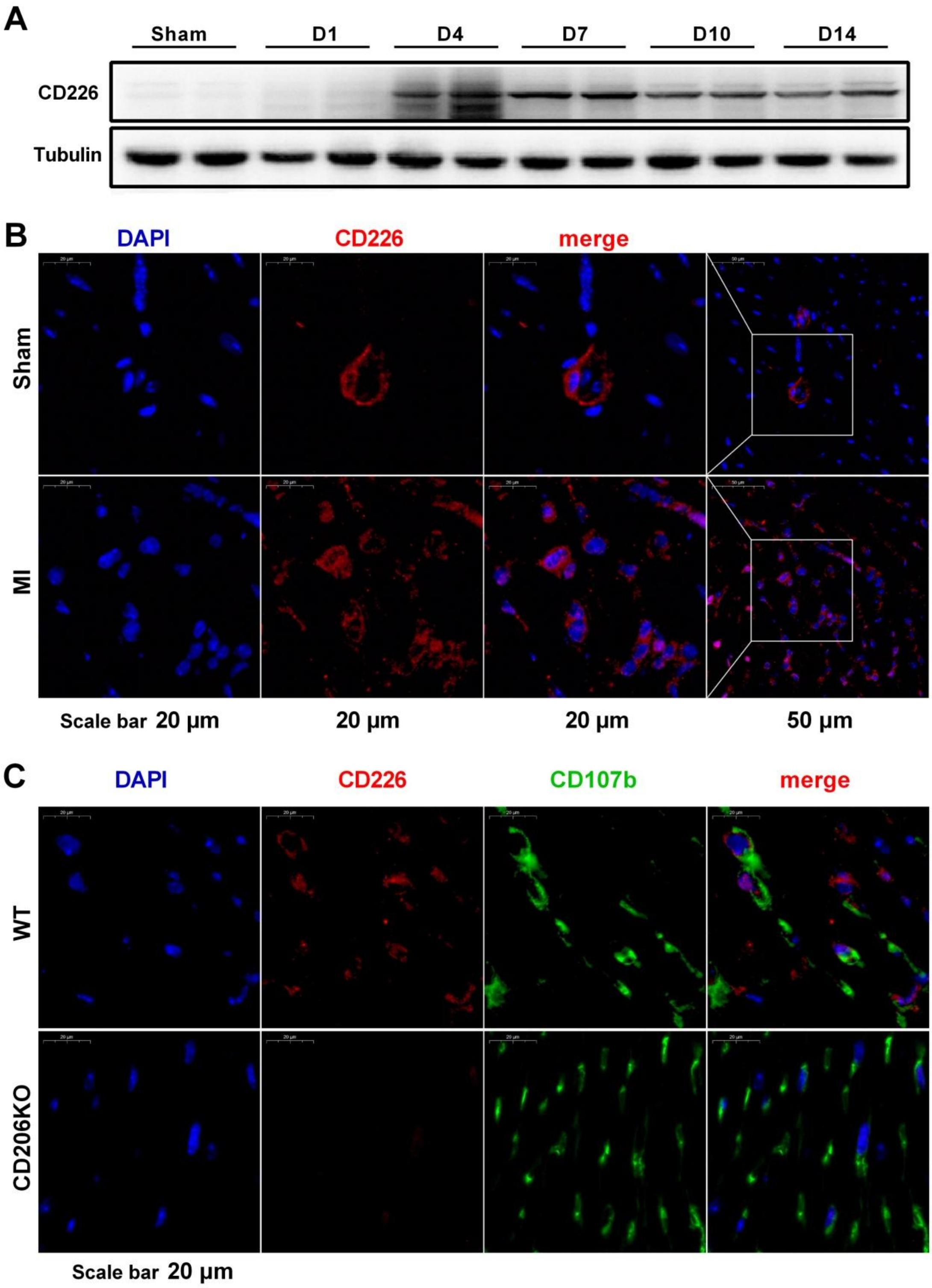

Figure S1 A, Representative immunoblots showing CD226 expression in infarcted heart tissue at different time points after MI. **B**, Representative images of immunofluorescence staining for CD226 of WT hearts 7 days after sham surgery or MI. **C**. Representative images of immunofluorescence co-staining for CD226 (red) and Mac-3 (green) in the infarcted hearts of WT and CD226KO mice 7 days after MI. DAPI was used for nuclear staining (blue). Scale bars, 20 μm.

Figure S2

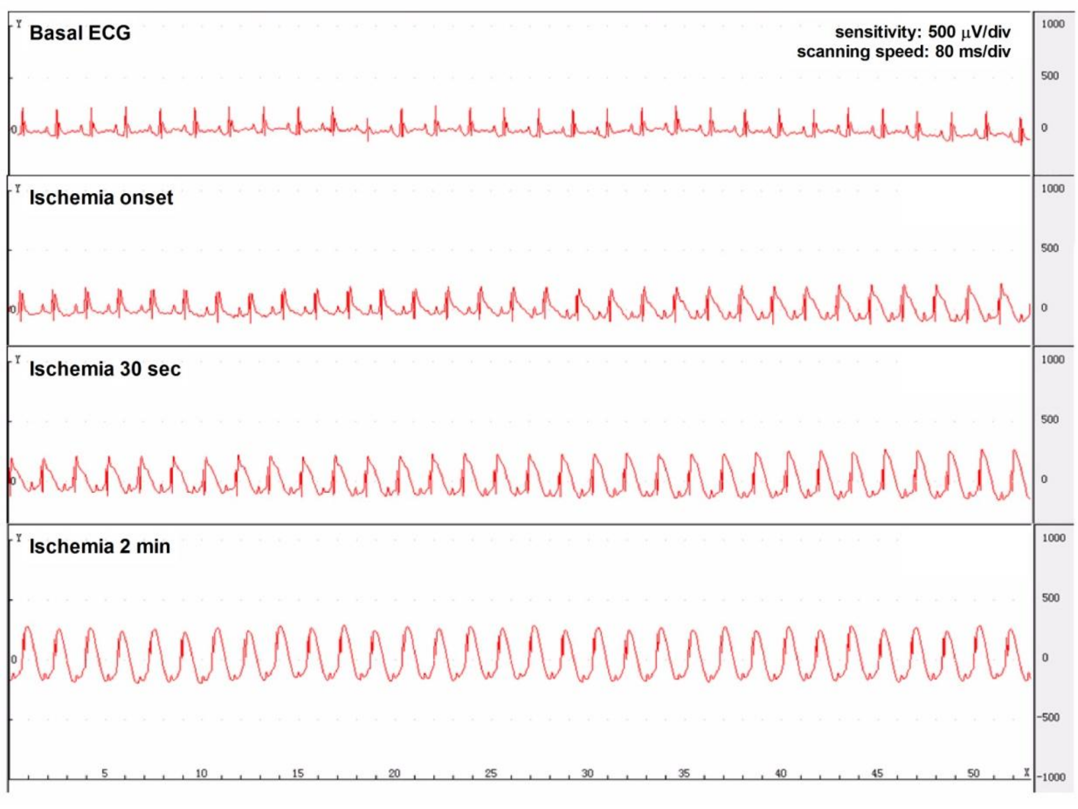

Figure S2 Representative electrocardiograph (ECG) tracing before and after LAD coronary artery ligation showing progressive ST-segment elevation, indicating transmural myocardial ischemia.

Figure S3

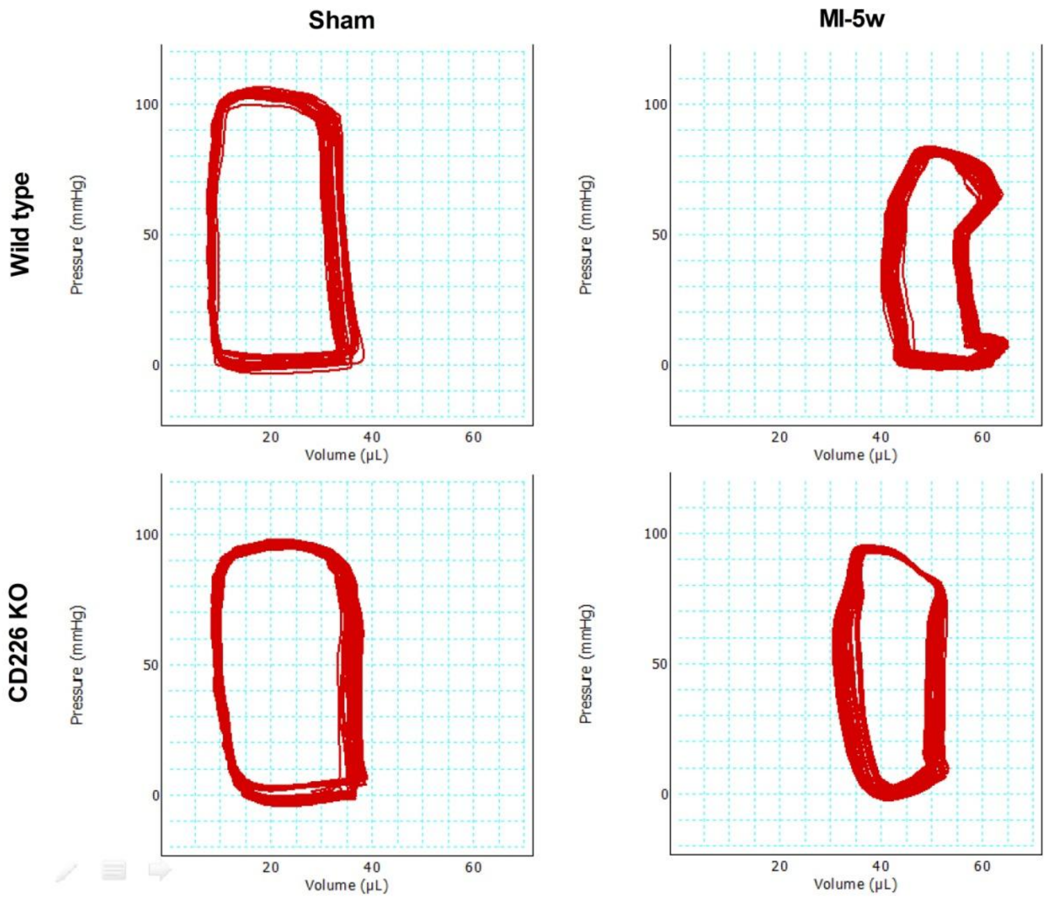

Figure S3 Representative LV pressure-volume loops acquired from WT and CD226 KO mice 5 weeks after MI or sham surgery using 1.4F Millar catheterization. MI resulted in depressed LV peak pressure and rightward shift of pressure-volume curve when compared to sham group. However, the rightward shift of pressure-volume curve was attenuated in the infarcted heart of CD226 KO mice compared with WT mice.

Figure S4

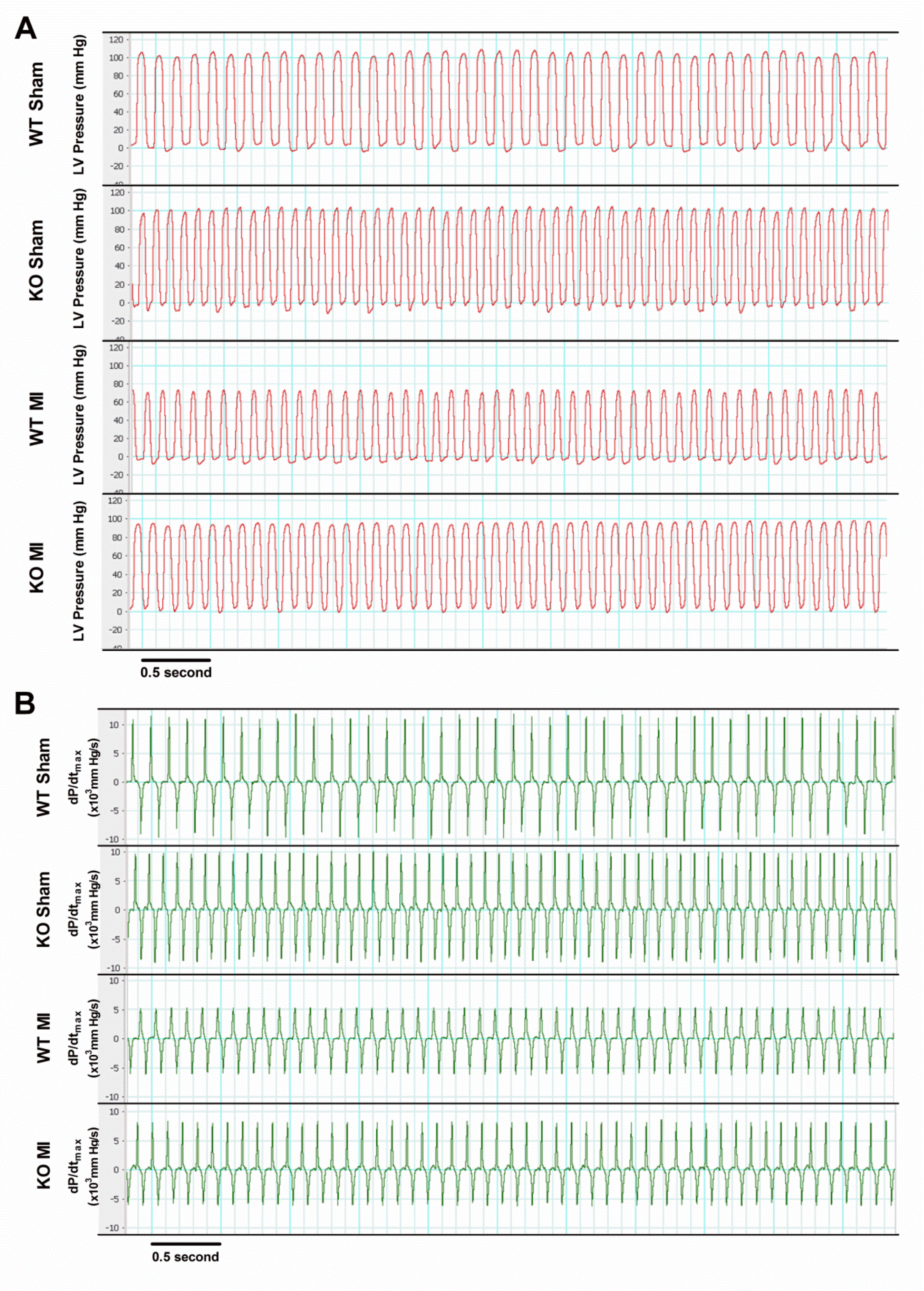

Figure S4 Representative hemodynamic recordings for LV pressure and $\pm dP/dt_{max}$ acquired from WT and CD226 KO mice 5 weeks after MI or sham surgery. There is no difference in the hemodynamic parameters between WT and CD226 KO mice subjected to sham operation. LVESP and $\pm dP/dt_{max}$ were depressed and LVEDP was elevated after MI, and CD226 KO mice displayed global improvement in these parameters compared with WT mice.

Figure S5

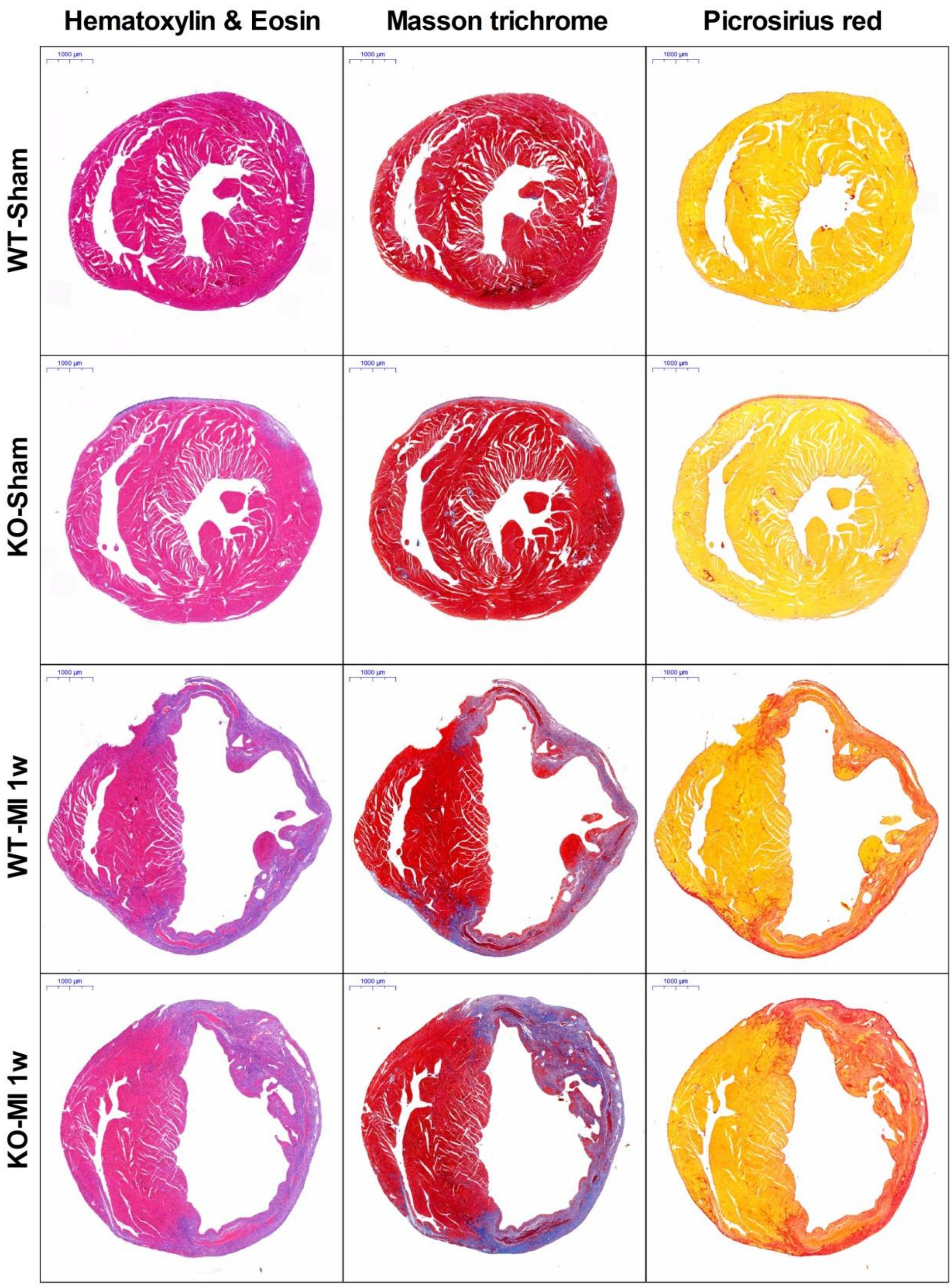

Figure S5 Representative images of hematoxylin and eosin (H&E), Masson trichrome and picrosirius red staining of serial heart sections from WT and CD226 KO mice 1 week after MI or sham operation. Scale bars, 1 mm.

Figure S6

A

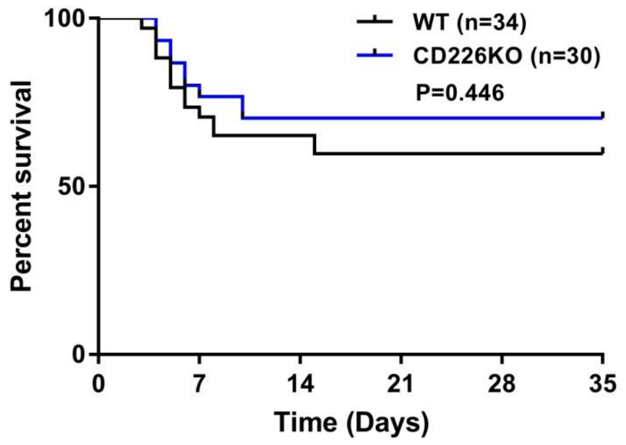

B

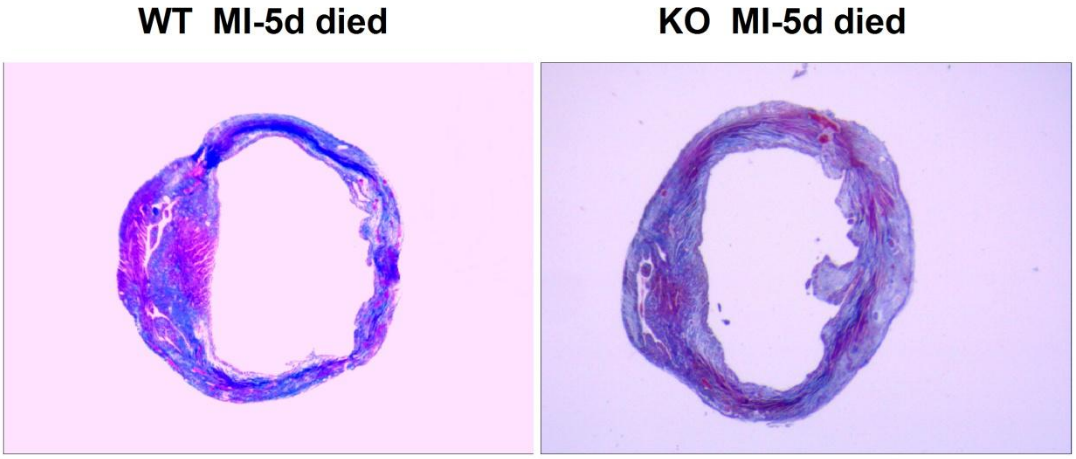

Figure S6 A, Comparison of survival curves between WT and CD226KO mice after 35 days of MI, and no significant differences in mortality were noted between the two groups. **B**, Overview of Masson trichrome staining of heart tissue sections from WT and CD226 KO mice died on day 5 after MI. WT mice exhibited thinner infarct wall thickness than CD226 KO mice.

Figure S7

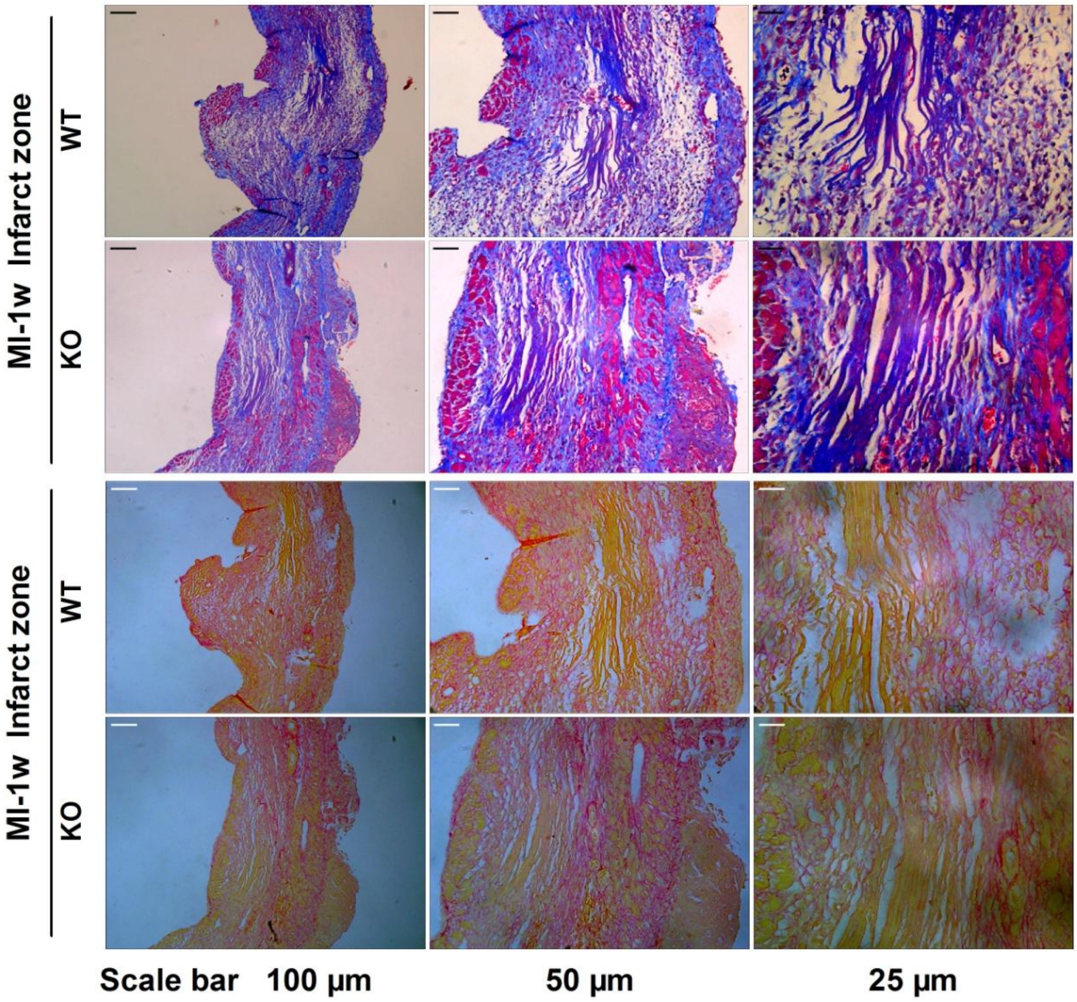

Figure S7 Masson trichrome and picrosirius red staining of serial sections showed more preserved myocardium and well-aligned collagen fibers in the infarct zone of CD226 KO mice 1 week after MI, in contrast to the infarct scar formation with loosely and fragmented collagen fibers in WT mice. Scale bars as indicated.

Figure S8

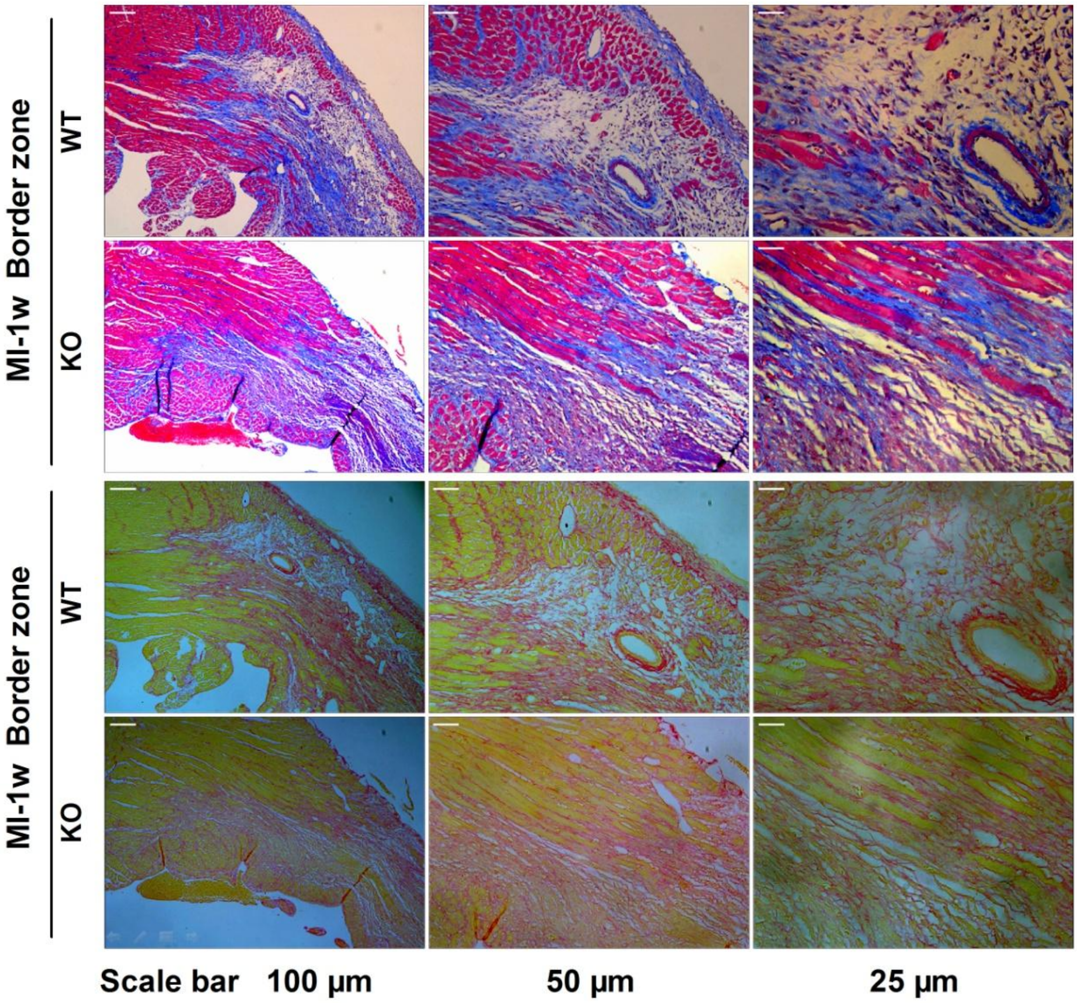

Figure S8 Masson trichrome and picrosirius red staining of serial sections show that WT mice exhibited loosely and fragmented collagen fibers and more fibrosis in the infarct border zone 1 week after MI, compared with CD226 KO mice. Scale bars as indicated.

Figure S9

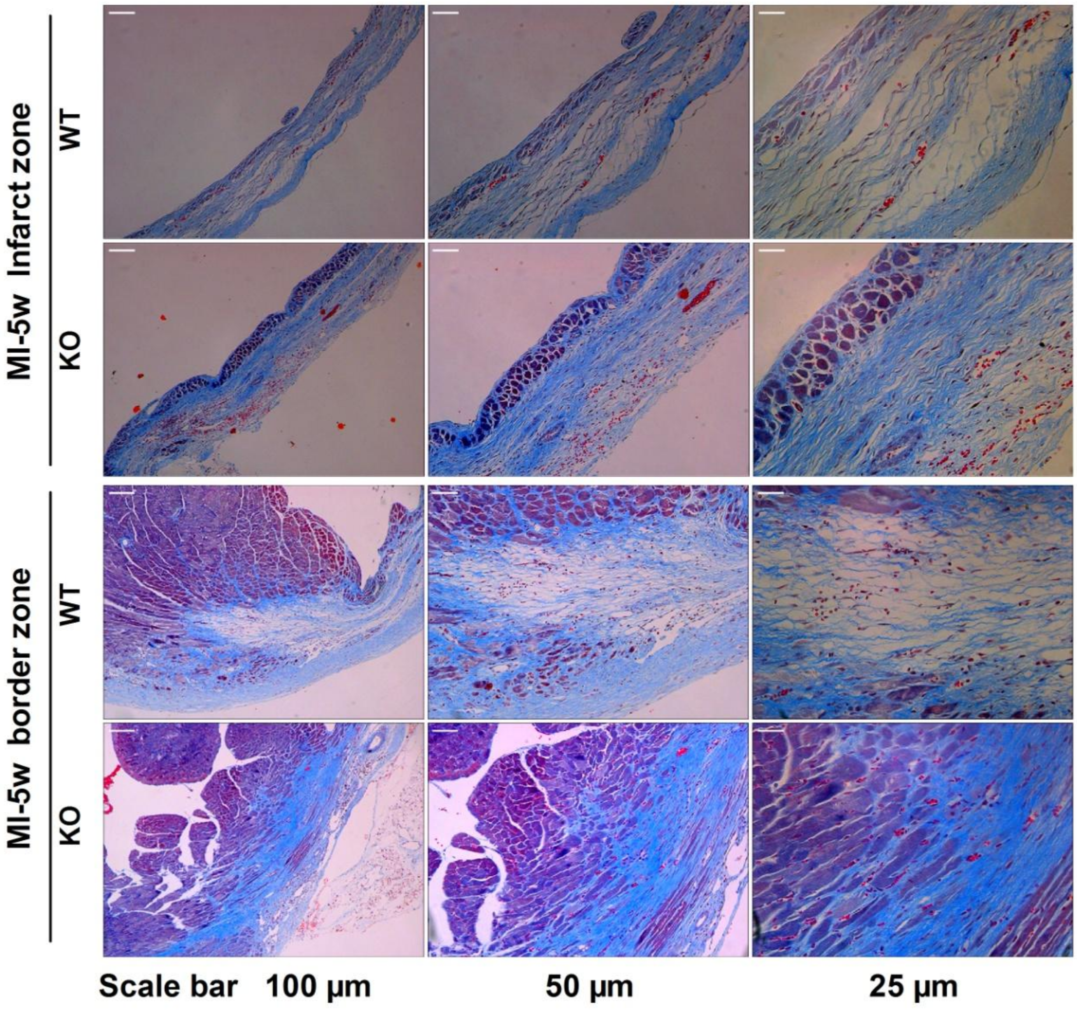

Figure S9 Representative masson trichrome staining images showing increased collagen deposition in the infarct zone, and attenuated interstitial fibrosis in the border zone of CD226 KO mice, when compared to WT mice 5 weeks after MI. More noticeably, fibrotic tissue of WT infarct border zone was mainly composed of loosely assembled and fragmented fibers, whereas, closely assembled and well aligned fibers were predominant in CD226 KO mice. Scale bars as indicated.

Figure S10

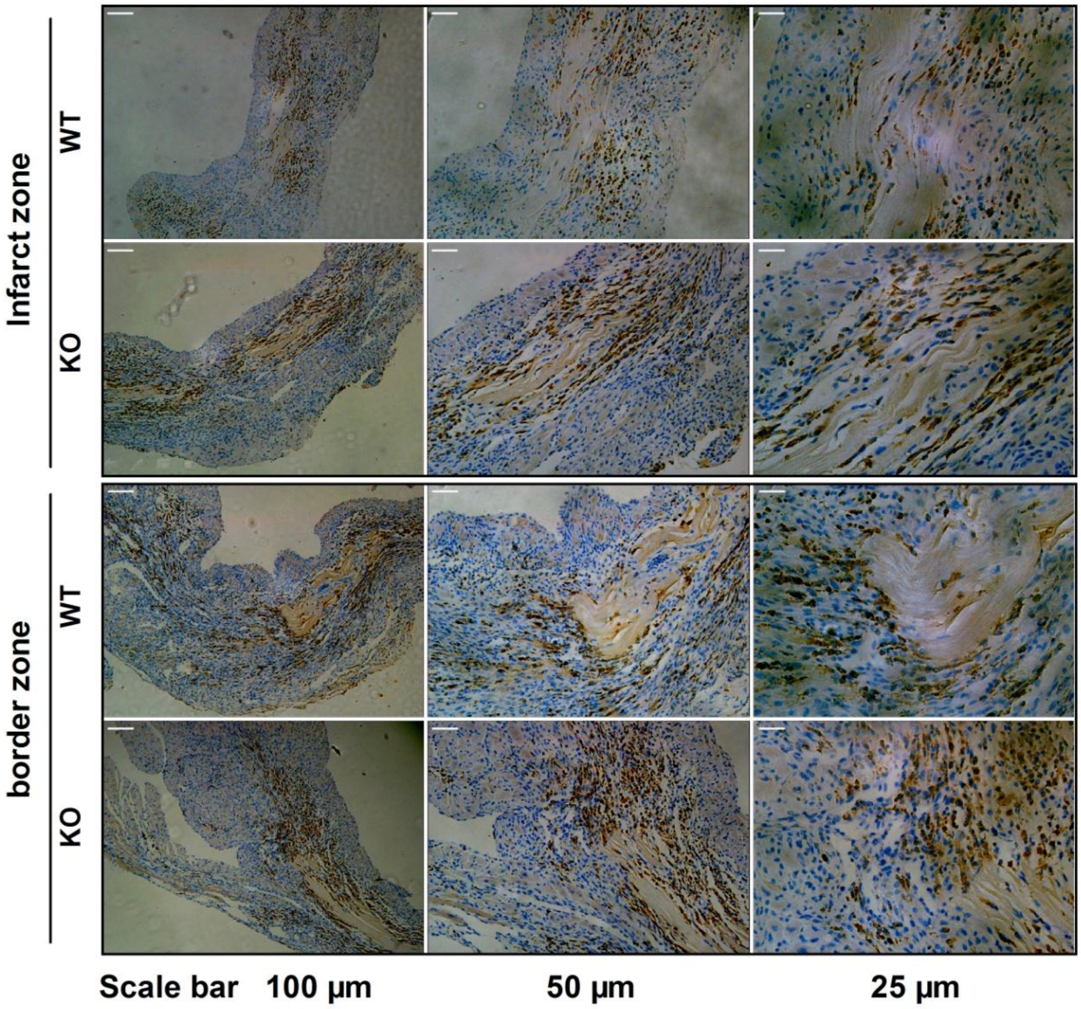

Figure S10 Immunohistochemical staining for $CD68^+$ macrophages in the infarct zone and border zone of WT and CD226 KO mice at day 7 after MI. Scale bars as indicated. Representative images from one of five samples are shown.

Figure S11

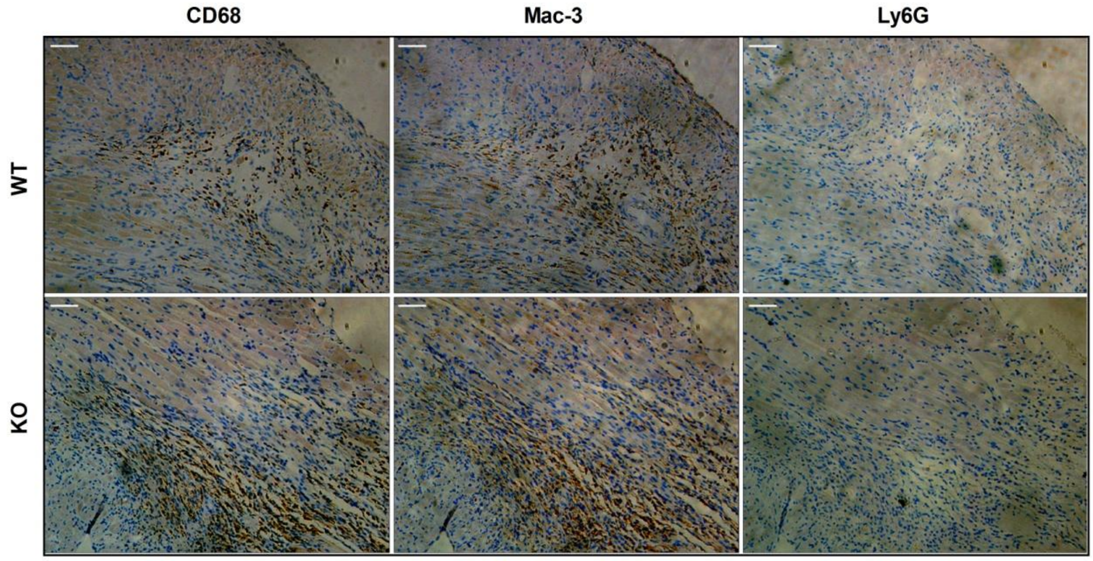

Figure S11 Immunohistochemical staining for CD68, Mac-3 and Ly6G in the infarct border zone of serial sections at day 7 after MI. Mac-3$^+$ macrophages showed a similar observation with CD68$^+$ macrophages. Ly6G$^+$ neutrophil infiltration was rarely observed in the ischemic heart at day 7 after MI. Scale bars, 50 μm.

Figure S12

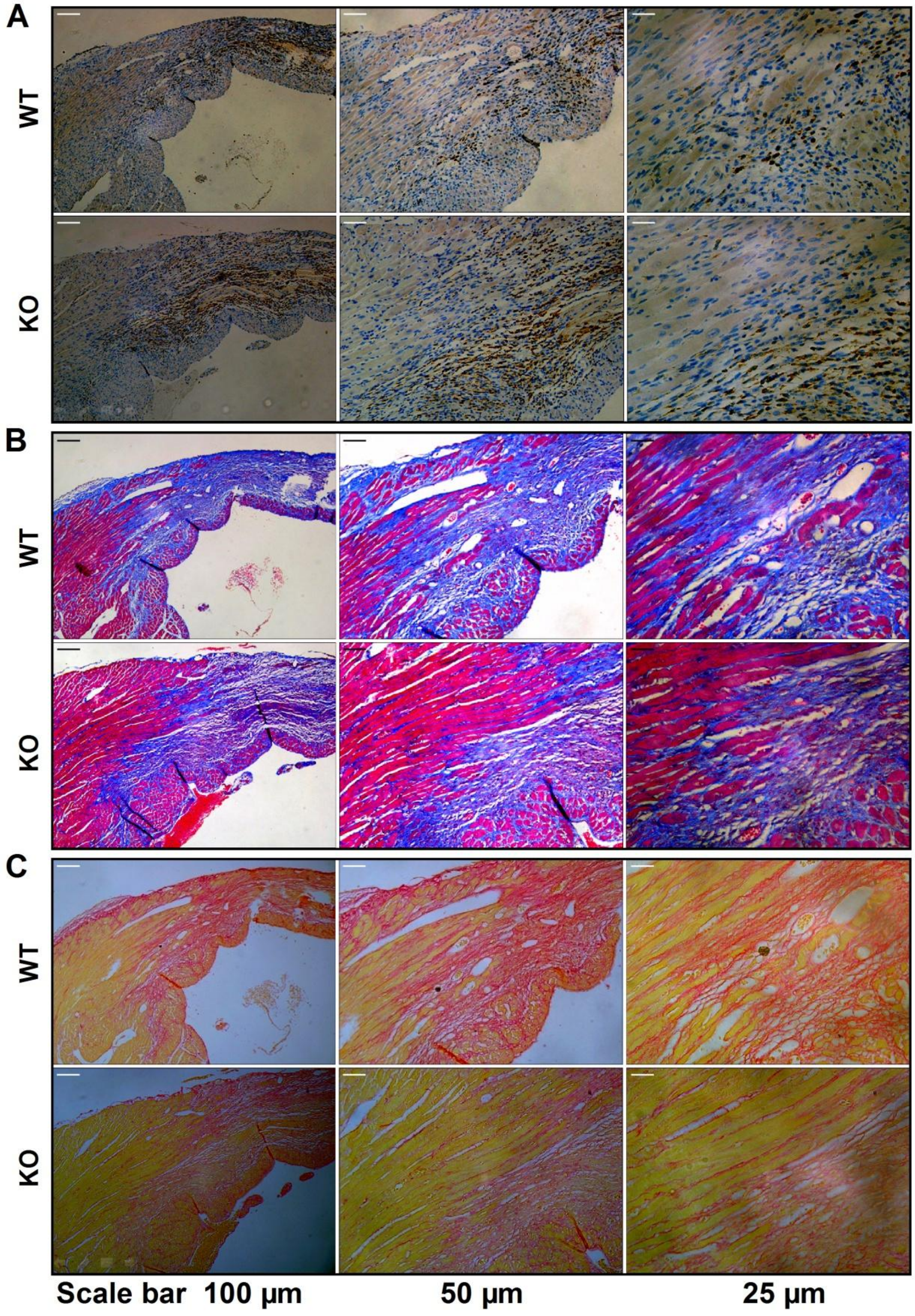

Figure S12 Serial heart sections were used for immunohistochemical CD68 staining, Masson trichrome and picrosirius red staining. Increased accumulation of $CD68^+$ macrophages corresponded to reduced interstitial fibrosis and well-aligned collagen fibers in the infarct border zone, as observed in CD226 KO mice. Scale bars as indicated. Representative images from one of five samples are shown.

Figure S13

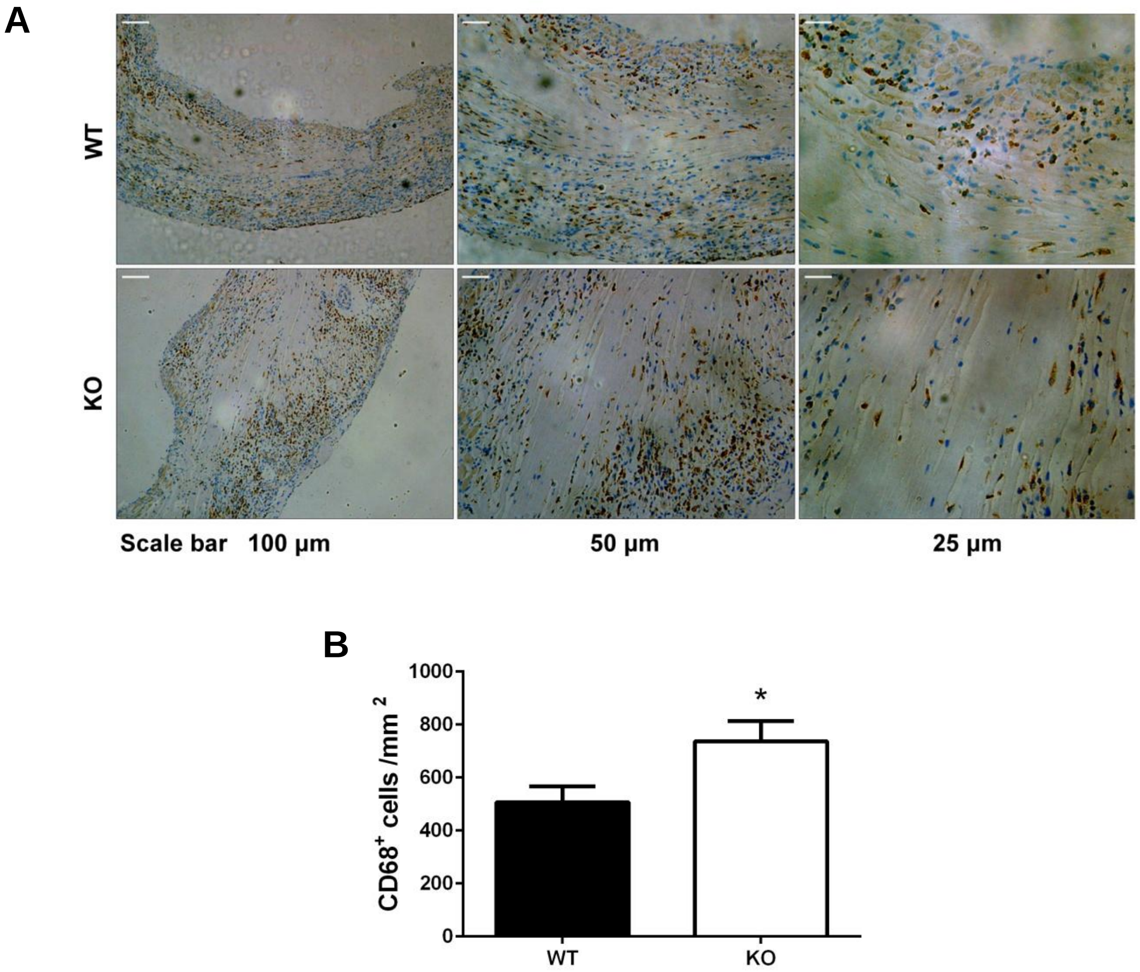

Figure S13 A, Representative images of immunohistochemical staining for CD68 in the infarcted hearts from WT and CD226 KO mice died on day 5 after MI. **B**, quantification data of CD68$^+$ macrophages and there was less CD68$^+$ macrophages infiltration in the infarcted hearts of WT mice died within one week after MI, compared with CD226 KO mice. * *P*<0.05, n=4 mice /each group.

Figure S14

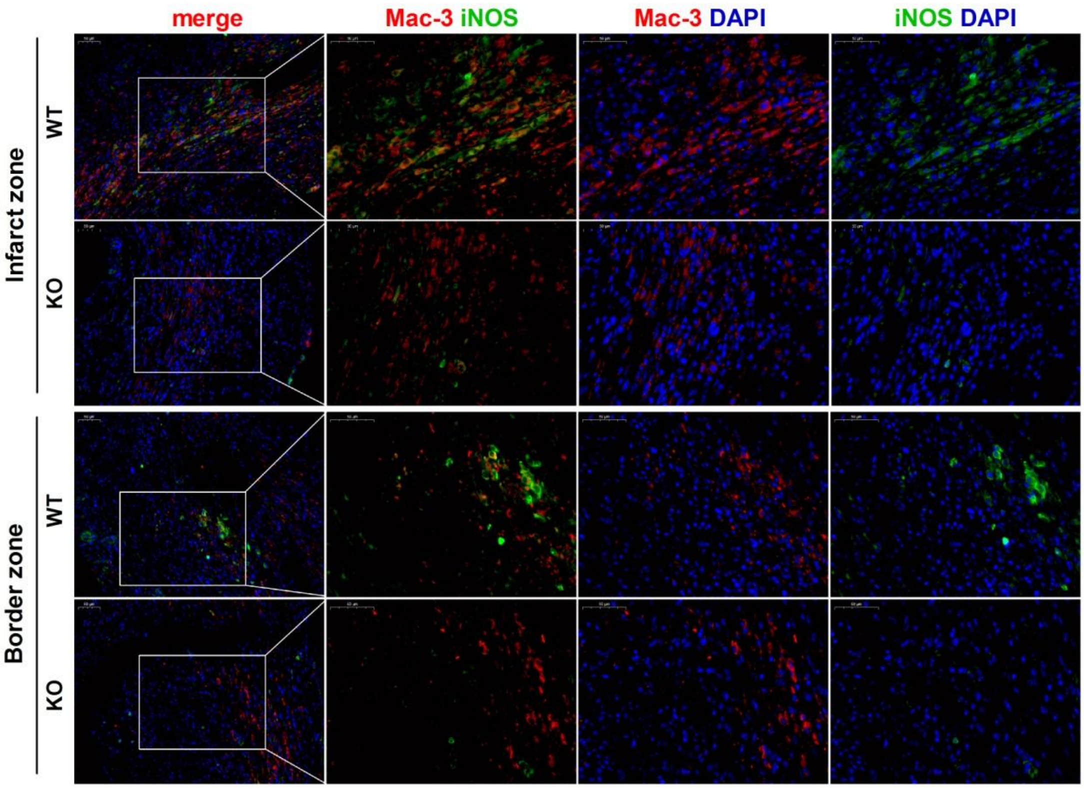

Figure S14 Immunofluorescence staining of Mac-3^{+} (red) iNOS^{+} (green) M1-like macrophages in the infarct and border area of WT and CD226 KO mice at day 7 after MI. DAPI was used for nuclear staining (blue). Scale bars as indicated. Representative images from one of four samples are shown.

Figure S15

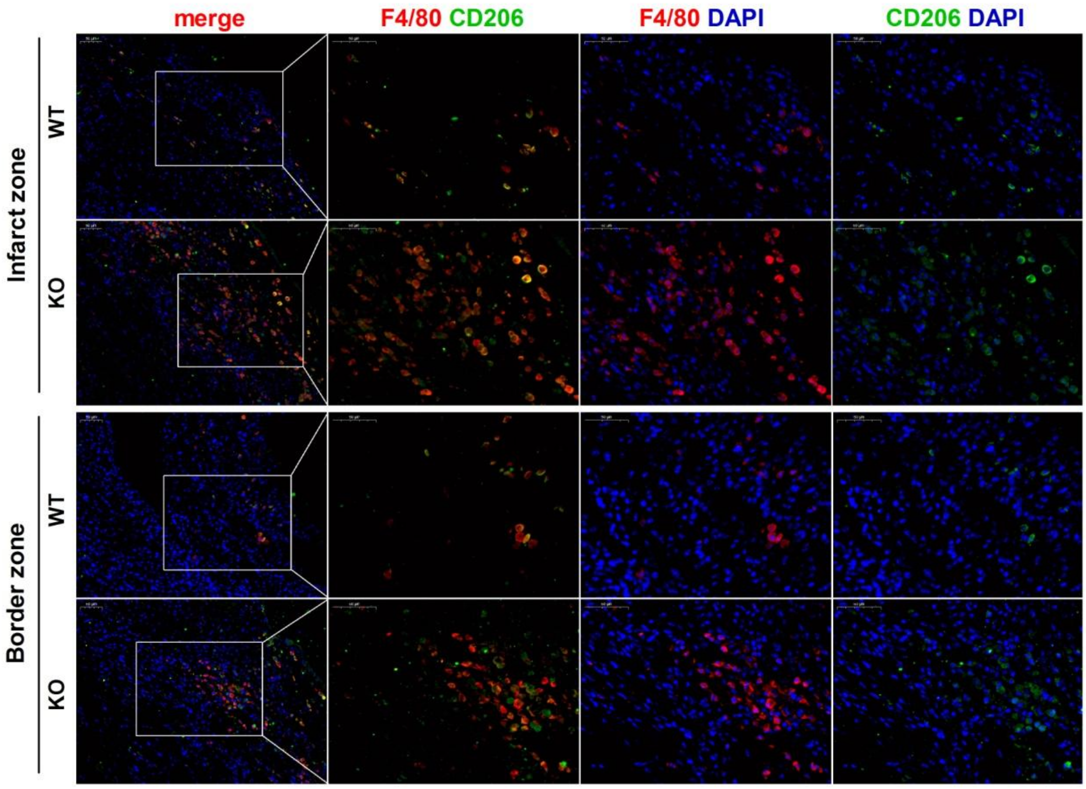

Figure S15 Immunofluorescence staining of F4/80$^+$ (red) and CD206$^+$ (green) M2-like macrophages in the infarct and border area of WT and CD226 KO mice at day 7 after MI. DAPI was used for nuclear staining (blue). Scale bars as indicated. Representative images from one of four samples are shown.

Figure S16

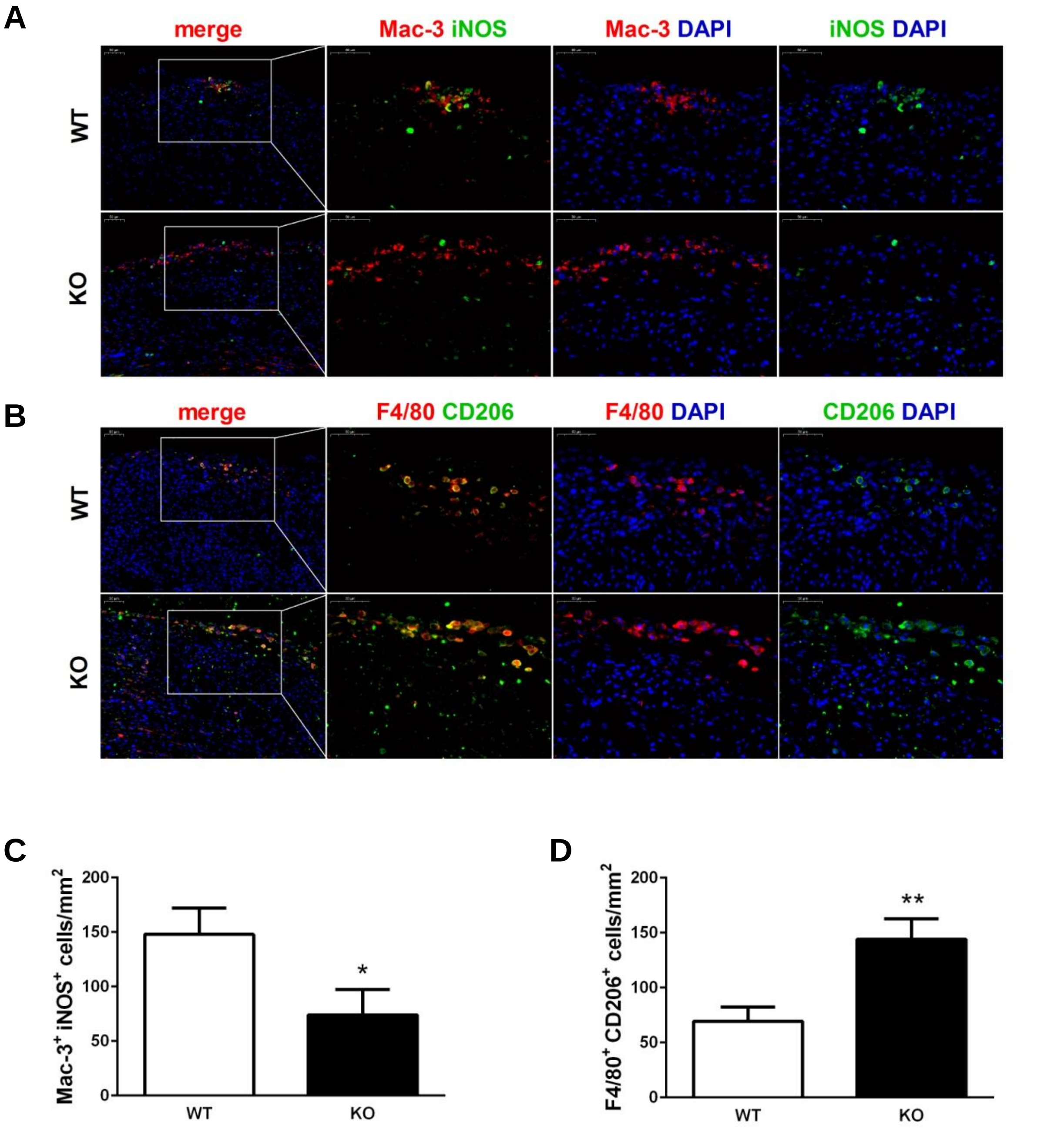

Figure S16 A-B, Immunofluorescence staining of Mac-3^+ (red) iNOS$^+$ (green) M1 macrophages (**A**) and F4/80^+ (red) CD206^+ (green) M2 macrophages (**B**) in the epicardium area of WT and CD226 KO mice at day 7 after MI. DAPI was used for nuclear staining (blue). Scale bars as indicated. **C-D**, quantification data of Mac-3^+ iNOS$^+$ macrophages (**C**) and F4/80^+ CD206^+ macrophages (**D**) in the epicardium area of infarcted heart. * P<0.05, ** P<0.01, n=5 mice /each group.

Figure S17

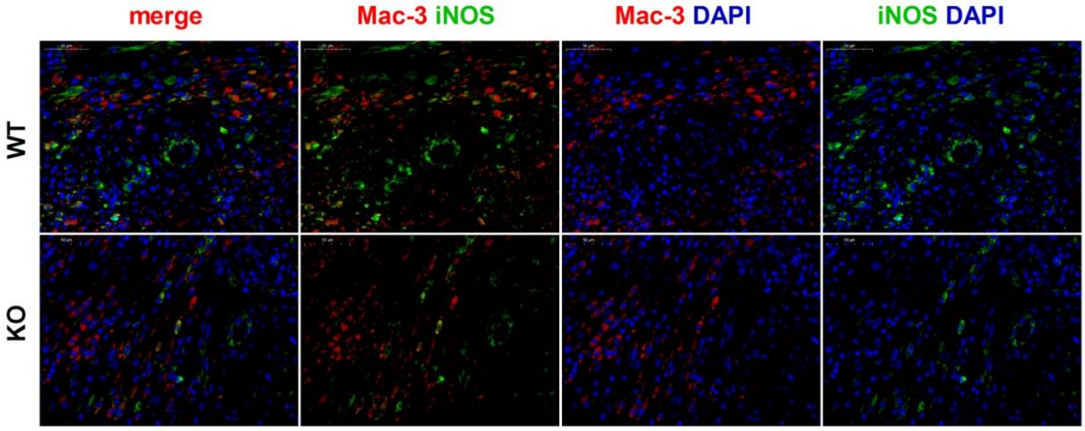

Figure S17 Immunofluorescence staining of Mac-3^{+} (red) iNOS^{+} (green) M1-like macrophages in the perivascular area of WT and CD226 KO mice 7 days after MI. DAPI was used for nuclear staining (blue). Scale bars as indicated. Representative images from one of four samples are shown.

Figure S18

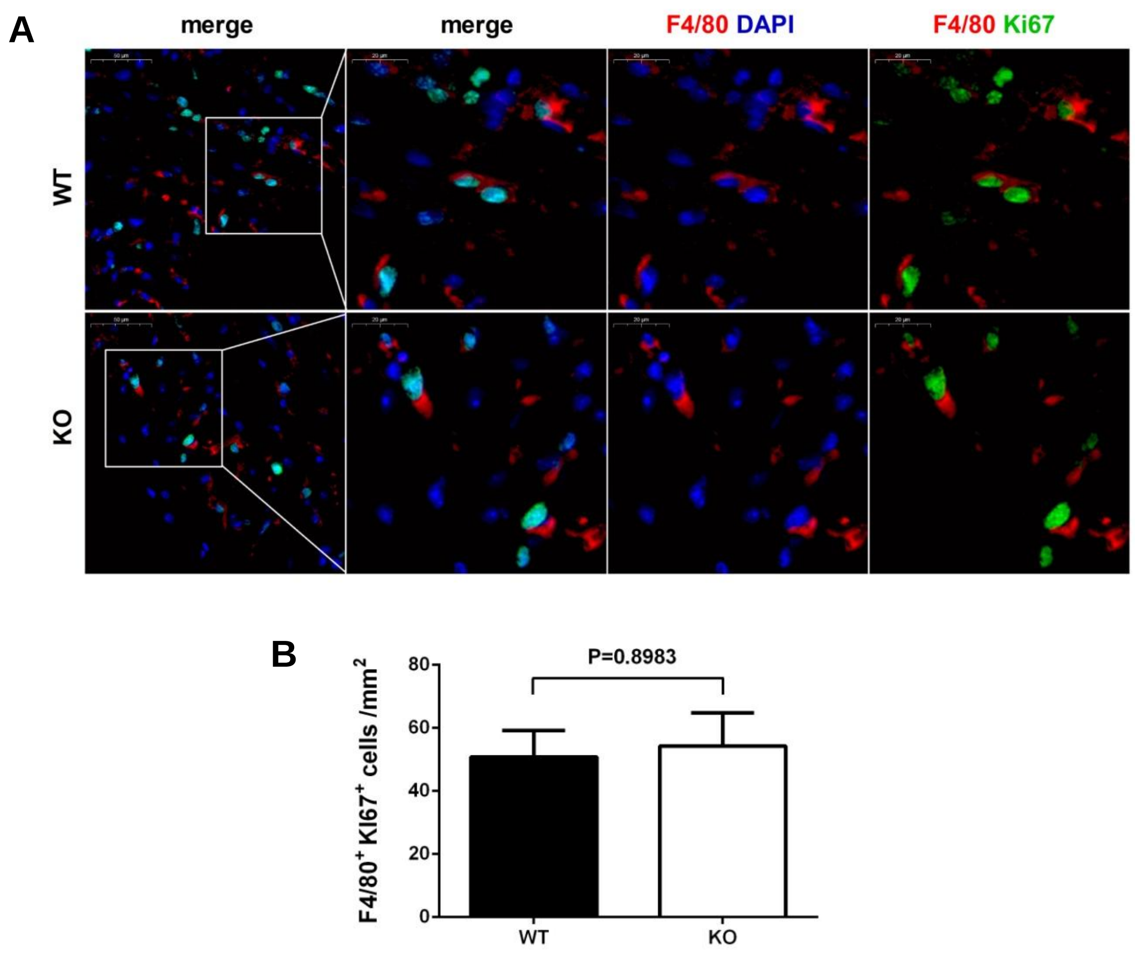

Figure S18 A, Immunofluorescence staining for F4/80 (red) and Ki67 (green) in the infarcted hearts of WT and CD226 KO mice 7 days after MI. DAPI was used for nuclear staining (blue). **B**, quantification data of F4/80$^+$ Ki67$^+$ proliferating macrophages in the infarcted hearts, and no significant difference between WT and CD226 KO mice was noted (n=5-6 mice /each group). Scale bars as indicated.

Figure S19

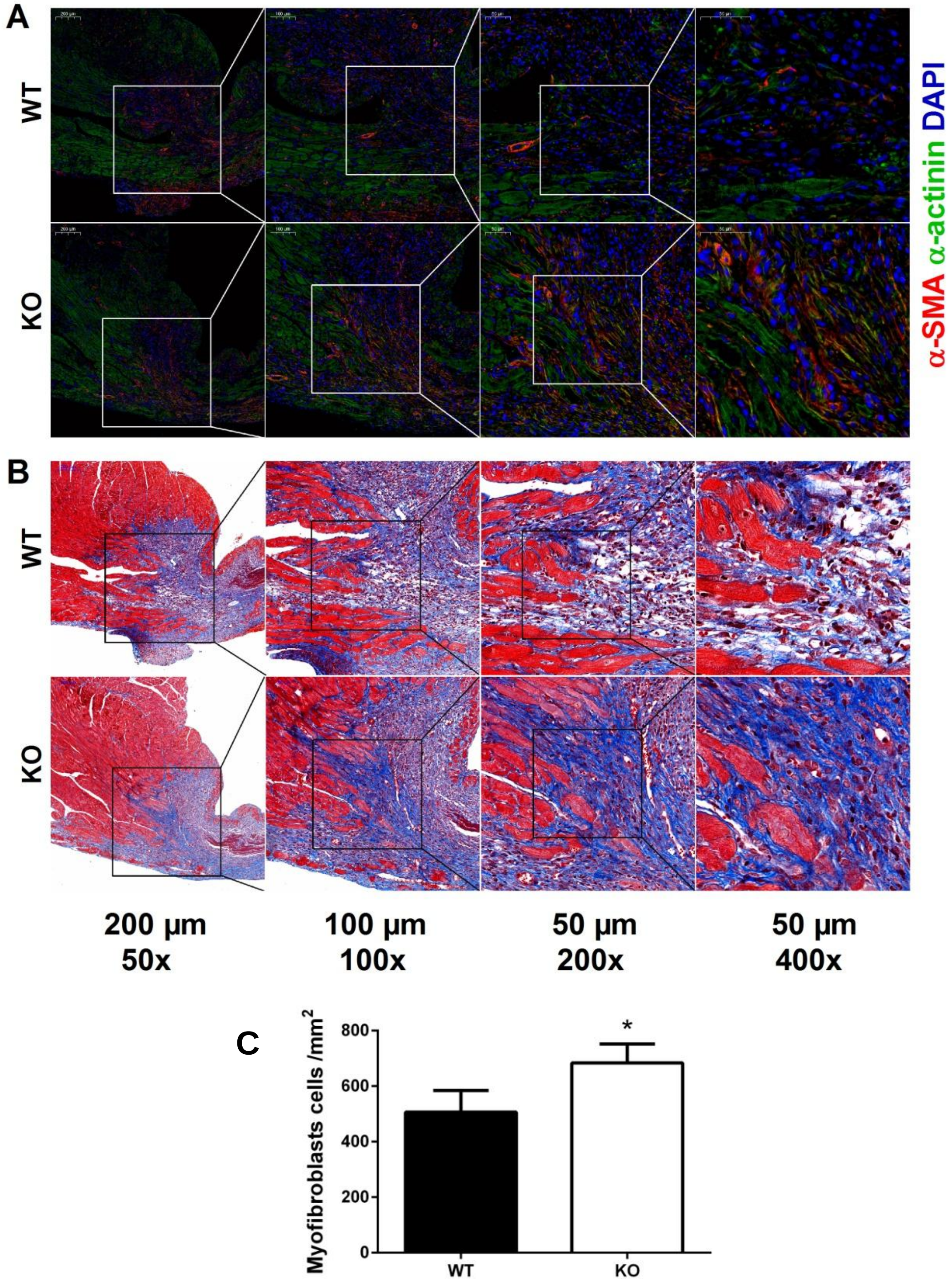

Figure S19 A, Immunohistochemical staining for α-smooth muscle actin (α-SMA) and α-actinin revealed an increase in the number of extravascular α-SMA$^+$ myofibroblasts in the infarct border zone of CD226 KO mice at day 7 after MI. **B**, Masson trichrome staining of serial heart sections at day 7 after MI revealed higher density of collagen deposition with well-aligned collagen fibers in the infarct zone of CD226 KO mice. Representative images from one of four samples are shown. **C**, quantification data of α-SMA$^+$ myofibroblasts in the infarct border zone of CD226 KO mice 7 days after MI.

Figure S20

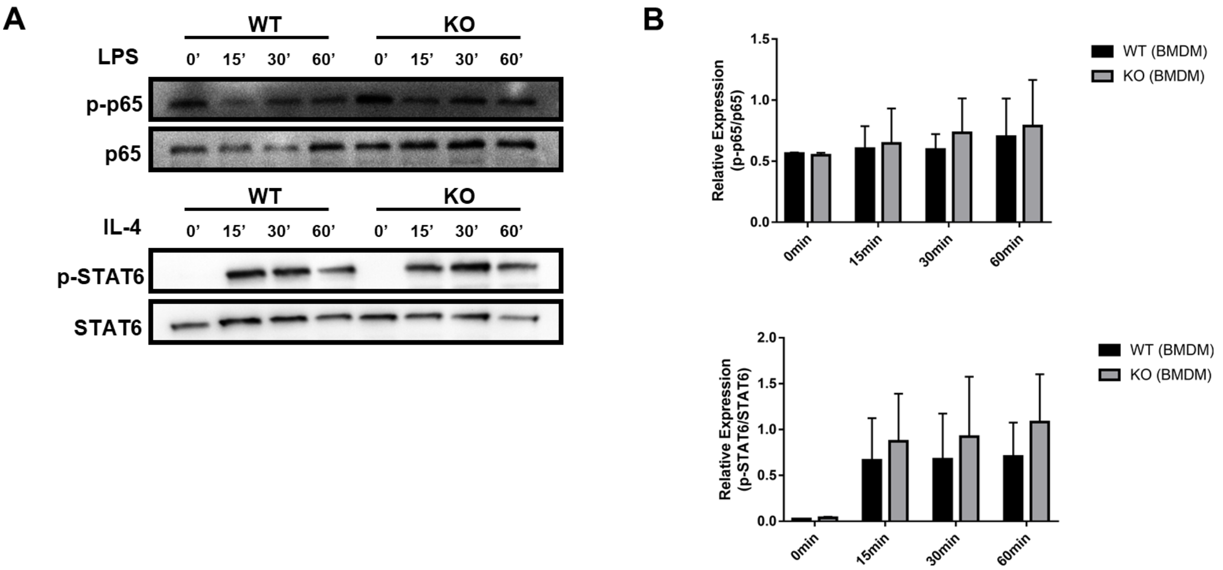

Figure S20 CD226 knockout facilitates STAT6 phosphorylation in BMDMs **A**, Representative images of Western blotting showed the NF-κB p65 (top) and STAT6 (bottom) phosphorylation of BMDMs from WT or KO mice which were stimulated with LPS or IL-4 respectively for 0 min, 15 min, 30 min and 60 min. **B,** Cumulative densitometry quantification for p-NF-κB p65 (top) and p-STAT6 (bottom) expression normalized to NF-κB p65 and STAT6, respectively. Results were repeated twice. Data are expressed as mean±SEM.

Figure S21

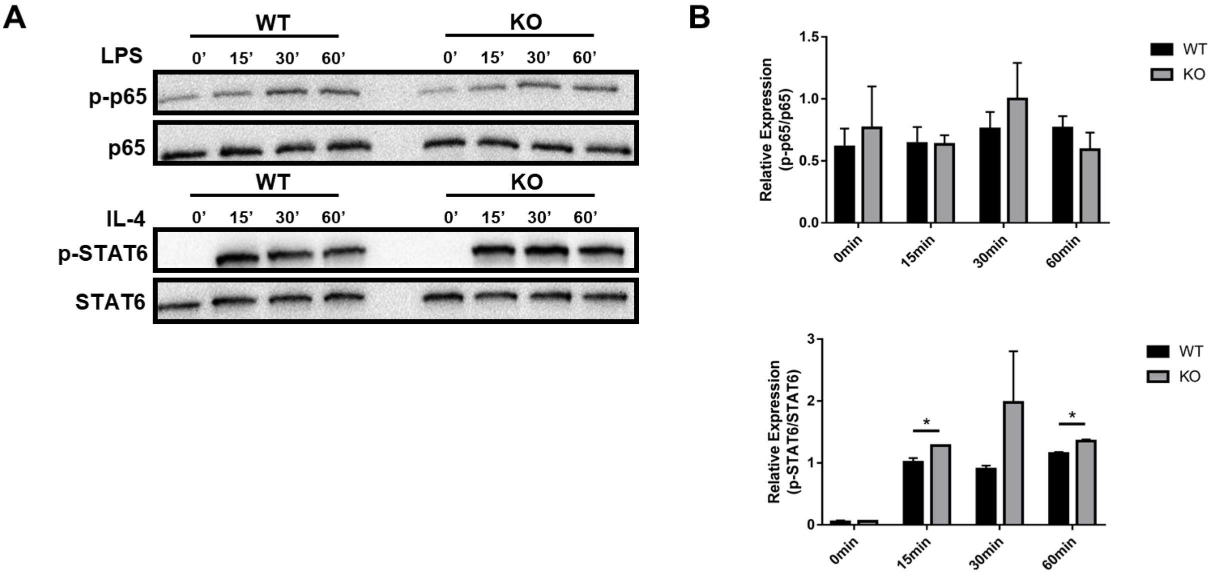

Figure S21 CD226 knockout increases STAT6 phosphorylation in peritoneal macrophages. **A**, Representative images of Western blotting showed the NF-κB p65 (top) and STAT6 (bottom) phosphorylation of peritoneal macrophages from WT or KO mice which were stimulated with LPS or IL-4 respectively for 0 min, 15 min, 30 min and 60 min. **B,** Cumulative densitometry quantification for p-NF-κB p65 (top) and p-STAT6 (bottom) expression normalized to NF-κB p65 and STAT6, respectively. Results were repeated twice. Data are expressed as mean ±SEM. * P<0.05vs corresponding WT.)

Table S1 Up-regulated proteins in the infarcted hearts of CD226 KO mice

**Accession**	**Gene Name**	**Description**	**Ration (KO/WT)**	**P value**
A0A0R4J1L2	Eef1d	Elongation factor 1-delta	1.270	0.038
P46664	Adss	Adenylosuccinate synthetase isozyme 2	1.311	0.016
Q9QXG2	Chm	Rab proteins geranylgeranyltransferase component A 1	1.313	0.009
Q544Z9	Cyb5a	Cytochrome b-5, isoform CRA_d	1.221	0.005
Q80ZI7	Igh	Igh protein	1.267	0.039
Q4FK74	Atp5d	ATP synthase, H+ transporting, mitochondrial F1 complex, delta subunit, isoform CRA_c	1.227	0.038
Q3UIZ8	Mylk3	Myosin light chain kinase 3	1.510	0.007
Q52L66	Ubl7	Ubiquitin-like 7 (Bone marrow stromal cell-derived)	1.471	0.010
Q9DCR2	Ap3s1	AP-3 complex subunit sigma-1	1.275	0.048
B2RSI0	Arhgap31	CDC42 GTPase-activating protein	1.429	0.014
P70255	Nfic	Nuclear factor 1 C-type	1.296	0.004
Q4VAG4	Rpl22	MCG12304	1.262	0.010
O54879	Hmgb3	High mobility group protein B3	1.241	0.041
Q5FW60	Mup20	Major urinary protein 20	1.696	0.013
Q3UGB8	Derl1	Derlin	1.534	0.010
Q99PM0	Mfap2	Microfibril-associated glycoprotein 1	1.540	0.042
Q9D5R3	Cep83	Centrosomal protein of 83 kDa	2.096	0.024

Table S1 Up-regulated proteins in the infarcted hearts of CD226 KO mice identified by iTRAQ proteomics. n= 3 per group, and each sample was the mixture of two infarcted hearts of the same group. The *P* value and WT/KO ratio of each differentially expressed protein was indicated (t test).

Table S2 Down-regulated proteins in the infarcted hearts of CD226 KO mice

Accession	Gene Name	Description	Ratio (KO/WT)	P value
A0A1D5RLR7	Mau2	MAU2 chromatid cohesion factor homolog	0.252	0.025
Q61941	Nnt	NAD(P) transhydrogenase, mitochondrial	0.287	0.002
Q0VEW4	Plp2	Proteolipid protein 2	0.639	0.005
Q8C650	Sept10	Septin-10	0.722	0.035
Q9CQV5	Mrps24	28S ribosomal protein S24, mitochondrial	0.683	0.041
Q8C7E4	Rnase4	Ribonuclease 4	0.803	0.046
Q8BTV2	Cpsf7	Cleavage and polyadenylation specificity factor subunit 7	0.744	0.023
Q3TSZ4	Galm	Aldose 1-epimerase	0.809	0.037
A0A0A6YVS2	Tmco1	Calcium load-activated calcium channel (Fragment)	0.823	0.035
Q0PD50	Rab8a	RAB8A, member RAS oncogene family, isoform CRA_a	0.750	0.010
P57784	Snrpa1	U2 small nuclear ribonucleoprotein A'	0.821	0.030
D3Z0N2	Sorbs2	Sorbin and SH3 domain-containing protein 2 (Fragment)	0.826	0.045
Q3TUQ5	Pnn	Pinin	0.775	0.000
A0A087WQ31	Clasp1	CLIP-associating protein 1	0.804	0.010
Q3TSI4	Gpat4	Putative uncharacterized protein (Fragment)	0.721	0.007
Q8VGJ1	Olfr796	Olfactory receptor	0.755	0.004
Q9D1M4	Eef1e1	Eukaryotic translation elongation factor 1 epsilon-1	0.826	0.004
O08739	Ampd3	AMP deaminase 3	0.811	0.001
Q80X95	Rraga	Ras-related GTP-binding protein A	0.772	0.022
Q91VK2	Eef1d	Eef1d protein	0.785	0.036
E0CYV0	Pcmt1	Protein-L-isoaspartate O-methyltransferase	0.832	0.009

Table S2 Down-regulated proteins in the infarcted hearts of CD226 KO mice identified by iTRAQ proteomics. n= 3 per group, and each sample was the mixture of two infarcted hearts of the same group. The *P* value and WT/KO ratio of each differentially expressed protein was indicated (t test).

Table S3 Primers for real-time PCR in this study

Gene		Sequence（5'-3'）	Size (bp)
GAPDH	Forward	AGGTCGGTGTGAACGGATTTG	123
	Reverse	TGTAGACCATGTAGTTGAGGTCA	
IL-1β	Forward	GCAACTGTTCCTGAACTCAACT	89
	Reverse	ATCTTTTGGGGTCCGTCAACT	
IL-6	Forward	TAGTCCTTCCTACCCCAATTTCC	76
	Reverse	TTGGTCCTTAGCCACTCCTTC	
IL-10	Forward	GCTCTTACTGACTGGCATGAG	105
	Reverse	CGCAGCTCTAGGAGCATGTG	
IL-12b	Forward	TGGTTTGCCATCGTTTTGCTG	123
	Reverse	ACAGGTGAGGTTCACTGTTTCT	
TNF-α	Forward	CCCTCACACTCAGATCATCTTCT	61
	Reverse	GCTACGACGTGGGCTACAG	
iNOS	Forward	GTTCTCAGCCCAACAATACAAGA	127
	Reverse	GTGGACGGGTCGATGTCAC	
Arg-1	Forward	CTCCAAGCCAAAGTCCTTAGAG	185
	Reverse	AGGAGCTGTCATTAGGGACATC	
CD206	Forward	CTCTGTTCAGCTATTGGACGC	132
	Reverse	CGGAATTTCTGGGATTCAGCTTC	
YM1	Forward	CAGGTCTGGCAATTCTTCTGAA	197
	Reverse	GTCTTGCTCATGTGTGTAAGTGA	
Fizz1	Forward	CCAATCCAGCTAACTATCCCTCC	188
	Reverse	ACCCAGTAGCAGTCATCCCA	

Supplemental Methods

Mice

Mice with homozygous deletions of CD226 genes (*CD226*$^{-/-}$) on a C57BL/6 background were kindly provided by Professor Marco Colonna (Washington University). C57BL/6 mice (8 weeks old) were purchased from Nanjing Biomedical Research Institute of Nanjing University (Nanjing, China). CD226$^{-/-}$ (CD226 KO) mice were backcrossed to C57BL/6 strain, then propagated by CD226$^{+/-}$ × CD226$^{+/-}$ mating. Wild-type (WT) mice (*CD226*$^{+/+}$) used as control in our experiments are the littermates of CD226 KO mice. Mice were maintained in a specific pathogen-free room at Experimental Animals Center of Fourth Military Medical University. Experiments were conducted using WT and CD226 KO adult male mice at the age of 10–12 weeks. Numbers of mice used for each experiment are given in figure legends. Animals in each group were randomly assigned to sham operation and MI groups (LAD ligation). This study and all animal procedures were performed in accordance with the Guide for the Care and Use of Laboratory Animals (NIH, Bethesda, MD), and were approved by the Animal Care Committee of the Fourth Military Medical University.

Mouse model of MI surgery

Adult male CD226 KO and WT mice at the age of 10–12 weeks were subjected to permanent ligation of the left anterior descending (LAD) coronary artery as described previously.[1] Briefly, mice were anesthetized with pentobarbital sodium (50 mg/kg) and then intubated using a 20-gauge intravenous catheter with a blunt end and ventilated with air with a small-animal respirator (tidal volume, 0.3 ml; rate, 150 breaths/ minute; Harvard Apparatus, USA). The chest wall was shaved, and a left thoracotomy was performed in the fourth intercostal space. The left ventricle and the LAD coronary artery were visualized using a microscope and the LAD artery was permanently ligated with a 7-0 monofilament suture at 1-2 mm distal to its emergence from under the left atrium. Infarction was confirmed by the presence of myocardial blanching in the ischemic area and ST segment elevation on the electrocardiogram. The thoracotomy was closed with 5-0 silk sutures. The endotracheal tube was removed once spontaneous respiration resumed, and animals were placed on a warm pad maintained at 37 ℃ until they were completely awake. Sham-operated animals underwent the same procedure without coronary artery ligation. Mice that did not survive the recovery from anesthesia and mice that died within 24 hours after surgery were excluded from the experiment.

Echocardiography and hemodynamics

Serial transthoracic echocardiographic measurements at baseline, 1 week, and 5 weeks after MI surgery was performed with a VEVO 2100 platform (Visual Sonics, Toronto, Canada) as described previously.[2] Mice were anesthetized by inhalation of isoflurane (1–2%) in a 100% oxygen mix, and heart rate was maintained at approximately 400-500 bpm in all mice during the echocardiographic examination to minimize data deviation. M-mode tracings were recorded through the anterior and posterior LV walls at the papillary muscle level to measure LV end-diastolic dimension (LVEDD) and LV end-systolic dimension (LVESD). The ejection fraction was calculated with the formula: [(LVEDV−LVESV)/LVEDV]×100, where EDV is end-diastolic volume and ESV is end-systolic volume. All the echocardiographic images were analyzed using Vevo 2100 software. The measurements represented the average of 6 selected cardiac cycles from at least 2 separate scans performed in random-blind manner.

Hemodynamic measurements and pressure-volume loop analysis

Invasive cardiac catheterization studies were performed 5 weeks after coronary artery ligation, using a 1.4 F pressure-volume conductance catheter (SPR-839, Millar Instruments) as described previously.[3] Mice were anesthetized with intraperitoneal injection of pentobarbital sodium (50 mg/kg) with spontaneous respiration. The

catheter was inserted into the right carotid artery and guided into the LV. Hemodynamic and volume parameters including Heart rate, LV end-systolic pressure, LV end-diastolic pressure, maximum rate of isovolumic pressure development ($\pm dP/dt_{max}$), LV end-diastolic volume (LVEDV) and LV end-systolic volume (LVESV) were measured and analyzed using a Power Lab System (AD Instruments Inc.). Pressure-volume loop data recorded at steady-state and during injection of hypertonic saline were analyzed for the calibration of parallel conductance volume (Vp). LV volume was calculated for each mouse from conductance volume corrected by the relative Vp. No less than fifty sequential beats were averaged for each measurement. At the end of the experiments, anaesthetized animals were sacrificed by cervical dislocation. Hearts were rapidly excised and prepared for histological analysis and protein extraction.

Morphometric analysis

Heart tissues were fixed in 4% paraformaldehyde, embedded in paraffin, and cut into 5-μm-thick sections. Sections were stained with hematoxylin and eosin, Masson trichrome and picrosirius red to determine the infarct size, collagen volume fraction and morphological effects. Infarct size was represented by scar circumference expressed as the ratio of scar circumference to the total LV circumference, including the septum, as described previously.[1] The wall thickness of the infarct scars at the papillary muscle and apical levels were measured as well. Cardiac fibrosis in infarct border zone was analyzed on Masson trichrome- or picrosirius red-stained sections, and collagen volume fraction was calculated as the ratio of the total area of interstitial fibrosis to the total area of interest. Myocyte/fibrosis ratio in the left ventricular free wall were determined and expressed as the ratio of myocyte area to fibrosis area as previously reported.[4] Collagen content in the infarcted region was quantitatively assessed with ImagePro software and expressed as the percentage of the area of the infarct, as described previously.[5] All these parameters were assessed in 5 randomly chosen high-power fields (×200) in each section. Results from all slides obtained in the same heart were averaged, and counted as n=1.

Immunohistochemistry and immunofluorescence

Paraffin-embedded sections (5 μm) were deparaffinized and rehydrated followed by heat mediated antigen retrieval. For immunohistochemical analysis, sections were pretreatment with 0.3% hydrogen peroxide for 20 min to inhibit endogenous peroxidase activity. Subsequently, sections were blocked with 3% BSA or serum for 30 min and incubated with the following primary antibodies: rat anti-CD68 (Clone: FA-11, MCA1957, AbD Serotec), rat anti-Mac3 (M3/84, Catalog no. 550292, BD Pharmingen), rat anti-Ly6G (MA1-10401, Thermo fisher) overnight at 4 ℃. After rinsing with PBS, the sections were incubated with goat-anti rat horseradish peroxidase (HRP) conjugated secondary antibody (Goodbio technology, Wuhan, China) for 50 min at room temperature. The stainings were immediately visualized with DAB detection kit (K5007, DAKO, Denmark), and sections were counterstained with hematoxylin. Quantitative assessment was performed by counting the number of immunoreactive cells in the infarct area or the border area. For immunofluorescence staining, sections were incubated with the following primary antibodies: rabbit anti-CD226 antibody (ab212077, Abcam), rat anti-F4/80 (ab6640, Abcam), rabbit anti-CD206 (ab195191, Abcam), rat anti-Mac-3 (M3/84, Catalog no. 550292, BD Pharmingen), rabbit anti-iNOS (ab178945, Abcam), rabbit anti-α-SMA (ab5694, Abcam), mouse anti-α-actinin (ab9465, Abcam), rat anti-CD31 (ab56299, Abcam), rabbit anti-Ki67 (ab16667, Abcam). Then, the sections were incubated with the appropriate Alexa-Fluor-coupled secondary antibodies for 1 h at room temperature, and Nuclei were stained with DAPI (Goodbio technology, Wuhan, China). The sections were scanned using Pannoramic MIDI and Images were acquired with CaseViewer software (3D HISTECH). Quantitative analysis was assessed in 5 randomly chosen high-power (×200) fields in each section. Results were averaged, and counted as n=1.

Western blot analysis

For western blot analyses, left ventricles were further dissected into an infarct zone, a border zone and remote myocardium (the basal part of the interventricular septum). LV samples were lysed in an ice-cold RIPA buffer. Equal amounts of protein were mixed with sample loading buffer and under reducing conditions separated on SDS-polyacrylamide gel. Proteins were electro-transferred on PVDF membranes (0.2 μm, Bio-Rad). Blots were incubated with following primary antibodies overnight at 4 ℃. Rabbit monoclonal anti-CD226 antibody (Clone: EPR20710, ab212077, Abcam, MA, USA). The bands were detected using a chemiluminescence assay (ECL Plus, ECL, Amersham). The goat anti-rabbit α-tubulin and GAPDH secondary antibodies were purchased from Beyotime (Jiangsu, China).

Cell preparation and flow cytometry

Spleen was excised, minced with fine scissors, and passed through a 70-μm nylon mesh in PBS. Erythrocytes were excluded with red blood cell (RBC) lysis buffer (TIANGEN，Catalog no. RT 122-02) and single cell suspension in PBS was refiltered through a 70-μm nylon mesh to remove connective tissue. Peripheral blood was collected in heparinized (EDTA-coated) tubes and incubated at room temperature for 30 min to sediment erythrocytes. Erythrocytes were removed using RBC Lysis buffer before FACS. Single cell suspensions from spleen and peripheral blood were stained on ice for 30 min with the following antibodies: anti-mouse Ly-6C-FITC (clone HK1.4, Biolegend, catalog no. 128006), anti-mouse Ly-6G-PE (clone 1A8, Biolegend, catalog no. 127608), anti-mouse CD11c-PerCP/Cy5.5 (clone N418, Biolegend, catalog no. 117328), anti-mouse/human CD11b-APC (clone M1/70, Biolegend, catalog no. 101212). All antibodies were used at a dilution of 1:100 in cell staining buffer (Biolegend, catalog no. 420201). Monocytes were identified as $CD11b^{+}CD11c^{-}Ly6G^{-}Ly6C^{high\ or\ low}$. Neutrophils were identified as $CD11b^{+}Ly6G^{high}$. Macrophages were identified as $CD11c^{-}Ly6G^{-}F4/80^{+}Ly6C^{high\ or\ low}$. Data were acquired on BD FACSCalibur flow cytometer (BD Biosciences), and results were analyzed on FlowJo. The total number of each cell population per milliliter of blood was calculated by multiplying the total number of cells by the percentage of cells within each cell gate.

Bone marrow-derived macrophages (BMDM) and *in vitro* macrophages stimulation

The tibias and femora were flushed with precooled PBS containing 2% FBS, and bone marrow cells were collected and strained through a 100 μm-nylon mesh, followed centrifugation at 1200 rpm for 5 min and resuspended in RBC Lysis buffer, then cultured in DMEM medium (Gibco) with recombinant murine M-CSF (10 ng/mL, PeproTech, Catalog no. 315-02) for 7 days. The BMDM were stimulated with interferon (IFN)-γ (20 ng/mL, PeproTech, Catalog no. 315-05) and lipopolysaccharide (LPS, 100 ng/mL, Sigma-Aldrich, Catalog no. L4391) for different times (6 h, 12 h, 24 h) to prime the cells to M1 phenotype, or were stimulated with IL-4 (10 ng/mL, R&D, 404-ML) for different times (6 h, 12 h, 24 h) to prime the cells to M2 phenotype.[6] The cells were incubated with fresh media for the unstimulated negative controls. The cells were harvested for RNA isolation.

RNA isolation and quantitative real-time polymerase chain reaction (PCR)

Total RNA was extracted using the TRIzol Reagent (Invitrogen) according to the manufacturer's instruction. RNA levels were quantified using the NanoDrop ND-1000 Spectrophotometer (Thermo Scientific). Reverse transcription of equal RNA content (0.5 μg) was performed using the PrimeScript® RT Master Kit (Takara), and real-time quantitative PCR was conducted using SYBR® Premix Ex Taq™ⅠⅠ(Takara) in accordance with the manufacturer's protocol. The sequences of primer pairs were designed using Primer Premier 5.0 software and are described in Supplemental Table 3. The gene levels were normalized to the reference gene GAPDH and the data

were reported as 2-ΔCt values ±SEM.

Protein digestion, iTRAQ labeling, LC-MS/MS analysis and protein quantification analysis

Each sample was prepared with 30 μ L protein solution, and adjusted to the final concentration of was 100mM with DTT (Dithiotheritol), following heated in boiling water for 5 min and cooled to room temperature, 200 μL UA buffer was added, and centrifuged at 14000g for 15 min twice. Then the sample was alkylated using IAA (Iodoacetamide; 100mM IAA in UA) for 30 minutes in dark at room temperature, followed by adding UA buffer and dissolution buffer in succession and centrifugation. It was then incubated with trypsin buffer (4 μg Trypsin in 40 μL Dissolution buffer) at 37 ℃ for 16 hours. The filtrate was collected and peptide fragment was quantified with Nano Drop 2000. Digested peptides (100 μg) were labelled with iTRAQ reagents according to manufacturer's protocol (Applied Biosystems, USA). The labeled proteins were analyzed by nano LC-MS/MS using Q Exactive equipped with an Easy n-LC 1000 HPLC system (Thermo Scientific) by Genechem Company (Shanghai, China). The raw data and protein quantification data were analyzed with software Mascot 2.5 and Proteome Discovery version 2.1, and we set an arbitrary threshold of 1.2-fold to declare differences.

Statistical analysis

Data were analyzed with GraphPad Prism-5 statistic software. All values are presented as the mean ±SEM of n independent experiments. One-way ANOVA was conducted across all investigated groups first. Post hoc tests were then performed with Bonferroni correction. For the iTRAQ data analysis, comparisons between 2 groups were performed using unpaired 2-tailed Student's t-test. Values of $P < 0.05$ were taken as statistically significant.

All authors had full access to and take full responsibility for the integrity of the data. All authors have read and agree to the manuscript as written.

References:

1. Kaikita K, Hayasaki T, Okuma T, Kuziel WA, Ogawa H, Takeya M. Targeted deletion of CC chemokine receptor 2 attenuates left ventricular remodeling after experimental myocardial infarction. Am J Pathol 2004; 165:439-447.
2. Saxena A, Fish JE, White MD, Yu S, Smyth JW, Shaw RM, DiMaio JM, Srivastava D. Stromal cell-derived factor-1alpha is cardioprotective after myocardial infarction. Circulation 2008; 117:2224-2231.
3. Fraccarollo D, Berger S, Galuppo P, Kneitz S, Hein L, Schütz G, Frantz S, Ertl G, Bauersachs J. Deletion of cardiomyocyte mineralocorticoid receptor ameliorates adverse remodeling after myocardial infarction. Circulation 2011; 123:400-408.
4. Yi W, Sun Y, Yuan Y, Lau WB, Zheng Q, Wang X, Wang Y, Shang X, Gao E, Koch WJ, Ma XL. C1q/tumor necrosis factor-related protein-3, a newly identified adipokine, is a novel antiapoptotic, proangiogenic, and cardioprotective molecule in the ischemic mouse heart. Circulation 2012; 125:3159-3169.
5. Bujak M1, Kweon HJ, Chatila K, Li N, Taffet G, Frangogiannis NG. Aging-related defects are associated with adverse cardiac remodeling in a mouse model of reperfused myocardial infarction. J Am Coll Cardiol 2008; 51:1384-1392.
6. Pelegrin P, Surprenant A. Dynamics of macrophage polarization reveal new mechanism to inhibit IL-1beta release through pyrophosphates. EMBO J 2009; 28:2114-2127.
